# Partially automated whole-genome sequencing reanalysis of previously undiagnosed pediatric patients can efficiently yield new diagnoses

**DOI:** 10.1038/s41525-020-00140-1

**Published:** 2020-08-11

**Authors:** Kiely N. James, Michelle M. Clark, Brandon Camp, Cyrielle Kint, Peter Schols, Sergey Batalov, Benjamin Briggs, Narayanan Veeraraghavan, Shimul Chowdhury, Stephen F. Kingsmore

**Affiliations:** 1 Rady Children’s Institute for Genomic Medicine, San Diego, CA USA; 2 Diploid, Leuven, Belgium

**Keywords:** Molecular medicine, Genetic testing

## Abstract

To investigate the diagnostic and clinical utility of a partially automated reanalysis pipeline, forty-eight cases of seriously ill children with suspected genetic disease who did not receive a diagnosis upon initial manual analysis of whole-genome sequencing (WGS) were reanalyzed at least 1 year later. Clinical natural language processing (CNLP) of medical records provided automated, updated patient phenotypes, and an automated analysis system delivered limited lists of possible diagnostic variants for each case. CNLP identified a median of 79 new clinical features per patient at least 1 year later. Compared to a standard manual reanalysis pipeline, the partially automated pipeline reduced the number of variants to be analyzed by 90% (range: 74%-96%). In 2 cases, diagnoses were made upon reinterpretation, representing an incremental diagnostic yield of 4.2% (2/48, 95% CI: 0.5–14.3%). Four additional cases were flagged with a possible diagnosis to be considered during subsequent reanalysis. Separately, copy number analysis led to diagnoses in two cases. Ongoing discovery of new disease genes and refined variant classification necessitate periodic reanalysis of negative WGS cases. The clinical features of patients sequenced as infants evolve rapidly with age. Partially automated reanalysis, including automated re-phenotyping through CNLP, has the potential to identify molecular diagnoses with reduced expert labor intensity.

## Introduction

For patients with suspected genetic disorders that remain undiagnosed after genomic sequencing, diagnostic yield is improved by periodic reanalysis^1–7^. While guidelines for reanalysis of whole-genome sequencing (WGS) or whole-exome sequencing (WES) data for undiagnosed patients do not yet exist, a recent position statement by the American Society of Human Genetics underlined the ethical obligation that clinical diagnostic laboratories and research groups have to support periodic WGS/WES data reanalysis^8^. Upon reanalysis, new diagnoses are made due to ongoing advances that include the discovery of new disease genes, accumulation of classified variants in publications and public databases, improvements in bioinformatics analyses, and phenotypic evolution in children in whom the full manifestations of disease were not apparent at initial analysis^1,5–7,9^. Current Procedural Terminology codes and Medicare fee payments have been established for the reanalysis of both WES (81417) and WGS (81427) for unexplained constitutional or heritable disorders or syndromes. The reanalysis pipelines described to date include manual phenotyping and extensive variant assessment, both of which are costly in terms of expert time. Automating or de-skilling portions of expert reanalysis can ease this burden, but the diagnostic yield and estimates on the savings of limited genomic analyst and laboratory director resources of such an approach have not yet been demonstrated. Here we present one solution to the challenge of ongoing WGS reanalysis. This pipeline integrates phenotyping from electronic health records (EHRs) by clinical natural language processing (CNLP) and a phenotypically driven analysis pipeline devised to alleviate the burden of next-generation sequencing (NGS) interpretation during reanalysis.

## Results

### Clinical characteristics of the reanalysis cohort

The first 48 inpatient children with suspected genetic disorders who received negative WGS reports after manual analysis between July 2016 and April 2017 were selected for partially automated reanalysis, using the original VCF files generated for analysis. The average patient age at enrollment was 4.8 months (range 3 days to 18 years or 0.1 to 238 months; Table 1) and the average time between enrollment and reanalysis was 19 months (range: 16–23 months). There was an equal sex distribution (Table 1). 50% of patients were of Hispanic/Latino descent and 29% were Caucasian, with the remainder of patients identifying as Asian/Pacific Islander, African or African-American, and Other/unknown (Table 1). The high percentage of Hispanic/Latino patients is consistent with the population characteristics of San Diego County. Disease phenotypes were highly diverse. The most common primary presentations at admission were neurological (27%), multiple congenital anomalies (25%), hepatic (13%), and hematological (10%) (Table 1).

**Table 1:** Demographic and clinical characteristics of the reanalysis cohort.

Age at enrollment	Median: 5 months; range 0.1–238 months	
	<1 month	10 (21%)
	<6 months	27 (56%)
Age at reanalysis^a^	Median: 24 months; range 0.1–255 months	
	<6 month	4 (8%)
	<24 months	23 (49%)
Sex	Female	23 (48%)
	Male	25 (52%)
Race and ethnicity	Hispanic/Latino	24 (50%)
	Caucasian	14 (29%)
	Asian/Pacific Islander	3 (6%)
	African/African American	2 (4%)
	Other/unknown	5 (10%)
Primary system involved	Neurological	13 (27%)
	Multiple congenital anomalies	12 (25%)
	Hepatic	6 (13%)
	Hematological	5 (10%)
	Musculoskeletal	3 (6%)
	Pulmonary	3 (6%)
	Cardiac	2 (4%)
	Endocrine/biochemical	2 (4%)
	Gastrointestinal	2 (4%)

^a^Or at the age of exitus.

### Reanalysis pipeline and resulting diagnoses

**Fig. 1:**
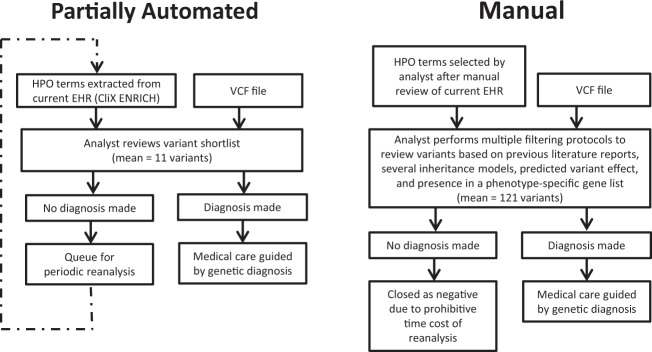
Comparison of partially automated and manual reanalysis pipelines. The partially automated reanalysis pipeline incorporates automated phenotype extraction from the EHR and variant shortlist generation.

The reanalysis pipeline incorporated automated re-phenotyping of patients at the time of reanalysis by CliX ENRICH and automated prioritization of a shortlist of variants by Moon based on imputed pathogenicity of all variants and rank ordering of genetic diseases associated with those variants based on similarity to the patient’s phenotype^10^ (Fig. 1, Supplementary Table 1). A major goal of automated shortlist generation within this reanalysis pipeline was to provide an easily interpretable dataset, reducing the need for expert time to be spent on reanalysis. The reanalysis variant shortlist contained a median of 11 variants (range: 5–29 variants), whereas this laboratory’s manual analysis/reanalysis pipeline contained a median of 121 variants (range: 91–168 variants) to review (Fig. 1). This represents a reduction of 90% of variants per case (range: 74–96%).

To assess whether the goal of providing an easily interpretable dataset was attained, all 48 shortlists were separately evaluated by an experienced analyst who produced a “truth set” and a novice analyst. The experienced analyst identified six potential new diagnoses (either one heterozygous variant or two variants in the compound heterozygous state), including two diagnoses that were reportable under Rady Children’s Institute for Genomic Medicine research protocols, and four possible diagnoses that were not reportable. The latter did not meet reporting requirements due to classification as variant(s) of uncertain significance (VUS) and, in some cases, an uncertain gene–disease relationship or unclear phenotypic matching with the patient (Table 2). Considering these six diagnoses as a truth set, the novice analyst identified 5 out of 6 diagnoses including both definite diagnoses (sensitivity = 0.83; the potential diagnosis in case 6046 was missed). Across the entire cohort, the novice analyst attained a specificity of 0.76, with 10 false positive diagnoses.

**Table 2:** Summary of cases with reported or possible diagnoses upon reanalysis.

Case	Phenotype	Potential diagnostic gene (RefSeq transcript ID)	Potential diagnostic variant(s)	Zygosity (inheritance)	Potential diagnosis	Classification	Outcome (reason not reported)	Resultant changes in medical care	Changes between initial analysis and reanalysis
6009	Small for gestational age and failure to thrive, congenital heart defects, bilateral ectrodactyly	*IGF2* (NM_001127598.1)	c.267C>A, p.(Cys89*)	Heterozygous (paternally phased de novo mutation)	Silver–Russell syndrome	P	Reported	Assessment of renal and hepatic function, blood pressure. Avoidance of metronizadole. Consideration of baclofen, carbidopa/levodopa	Correction of manual data labeling error. 2016 classification of this variant as pathogenic (GeneDx; ClinVar accession SCV000491643.1)
6033	Failure to thrive, intention tremor, ataxic gait, developmental regression, hypotonia, microcephaly, white matter abnormalities on MRI	*ERCC6* (NM_000124.3)	c.-15+3G>T; c.1583G>A, p.(Gly528Glu)	Heterozygous (paternal); heterozygous (maternal)	Cockayne syndrome type B	P; LP (after orthogonal functional testing)	Reported	Growth hormone treatment, hypoglycemia prevention, monitoring for premature adrenarche, maxillofacial assessment	Publication of c.1583G>A variant in Cockayne syndrome cohort (Calmels et al.^24^). Research-basis functional testing of patient fibroblasts
6046	Unprovoked cardiac arrest, sudden infant death syndrome, suspicion of long Q-T syndrome	*ANK2* (NM_001148.4)	c.1574C>T, p.(Ala525Val)	Heterozygous (maternal)	Long Q-T syndrome 4	VUS; VUS	Queue for periodic reanalysis (VUS)	N/A	2018 classification of this variant as a VUS in association with long Q-T syndrome (Invitae; ClinVar accession SCV000822853.1)
6062	Feeding difficulties, ketosis, mast cell activation disorder, hypotonia, joint hypermobility, fine motor delay, fatigue after exercise	*GAA* (NM_000152.4)	c.-32-13T>G; c.497C>T; p.(Thr166Ile)	Heterozygous (not maternal); heterozygous (maternal)	Glycogen storage disease II	P; VUS	Queue for periodic reanalysis (VUS, unclear phenotypic matching)	N/A	*GAA* variants reported to patient by GeneDx, following physician-requested analysis of clinical exome data for GSD genes. Subsequent functional testing of patient alpha glucosidase, with results in low-normal range
6068	Acute lymphoid leukemia in remission, rectal fistula, gallstones, fevers, clotting	*JAK2* (NM_004972.3)	c.2600G>A, p.(Arg867Gln)	Unknown (parental samples unavailable)	Leukemia susceptibility (novel gene/disease association)	VUS	Queue for periodic reanalysis (VUS, unclear gene–disease relationship)	N/A	Publication of this variant segregating in a kindred with thrombocytosis and progression to polycythemia vera in one affected (Maie et al.^25^)
6081	Arthrogryposis multiplex congenita, micrognathia, desaturations, parenchymal dysplasia, cortical dysplasia, bilateral clubfeet, suspected congenital knee dislocation, uterine growth restriction	*B3GALT6* (NM_080605.3)	c.122C>T, p.(Ala41Val); c.950C>T p.(Pro317Leu)	Heterozygous (maternal); heterozygous (paternal)	Ehlers–Danlos syndrome, spondylodysplastic type 2	VUS; VUS	Queue for periodic reanalysis (VUS, unclear phenotypic matching)	N/A	—

*P* Pathogenic, *LP* Likely Pathogenic, *VUS* Variant of Uncertain Significance.

The two reportable diagnoses, cases 6009 and 6033, were returned to referring physicians, resulting in changes in their patients’ medical care, while the four possible diagnoses were queued for further periodic reanalysis (Fig. 1, Table 2). Patient 6009 carried a de novo stop-gain variant in insulin-like growth factor 2 (somatomedin A), *IGF2* c.267C>A, p.Cys89*, which was phased by Sanger sequencing to the paternal allele using a nearby informative single-nucleotide polymorphism (SNP; rs3213225). Since *IGF2* is an imprinted gene that is expressed exclusively from the paternal allele, phasing was crucial for reporting a diagnosis of Silver–Russell syndrome (OMIM #180860). This variant was not identified during initial analysis due to a manual error in patient data handling. This diagnosis was expected to change the patient’s clinical care, indicating targeted monitoring for hypoglycemia, premature adrenarche and maxillofacial abnormalities, and potentially treating with growth hormone (Table 2).

Patient 6033 carried two variants in trans in Excision Repair Cross-Complementing Group 6 (*ERCC6)*. They were a missense variant, c.1583G>A, p.Gly528Glu and an intronic variant, c.−15+3G>T, both of which were classified as VUSs at initial analysis and thus not reportable under the institutional review board (IRB) protocol for this study. During the period between analysis and reanalysis, *ERCC6* c.1583G>A was reported in a patient with Cockayne syndrome, type B (OMIM #135540), changing its classification to Likely Pathogenic^11^. In parallel, research functional testing of unscheduled DNA synthesis from proband fibroblasts confirmed an impairment of *ERCC6*. This functional data changed the variant classifications to Pathogenic (c.1583G>A) and Likely Pathogenic (c.−15+3G>T), rendering them reportable under our IRB protocol. This diagnosis was also expected to inform clinical care, indicating avoidance of metronizadole, monitoring of renal and hepatic function and blood pressure, screening for cataracts and strabismus, increased sun protection, and potentially treating with baclofen or carbidopa/levodopa (Table 2).

Another benefit of periodic reanalysis of negative cases is the incorporation of improvements in bioinformatics analyses. Copy number variants (CNVs) were not evaluated at the time of original analysis or at initial reanalysis. In a separate effort, we evaluated CNVs in these 48 cases and found two potential diagnoses: an inherited intragenic KAT6B deletion in case 6033, classified as Likely Pathogenic and thought to co-contribute to the patient’s phenotype (see Table 2), and a de novo 3.5 MB deletion at 12q21.33-q22 in case 6034, classified as Likely Pathogenic.

### CNLP-based phenotyping and downstream variant prioritization

**Fig. 2:**
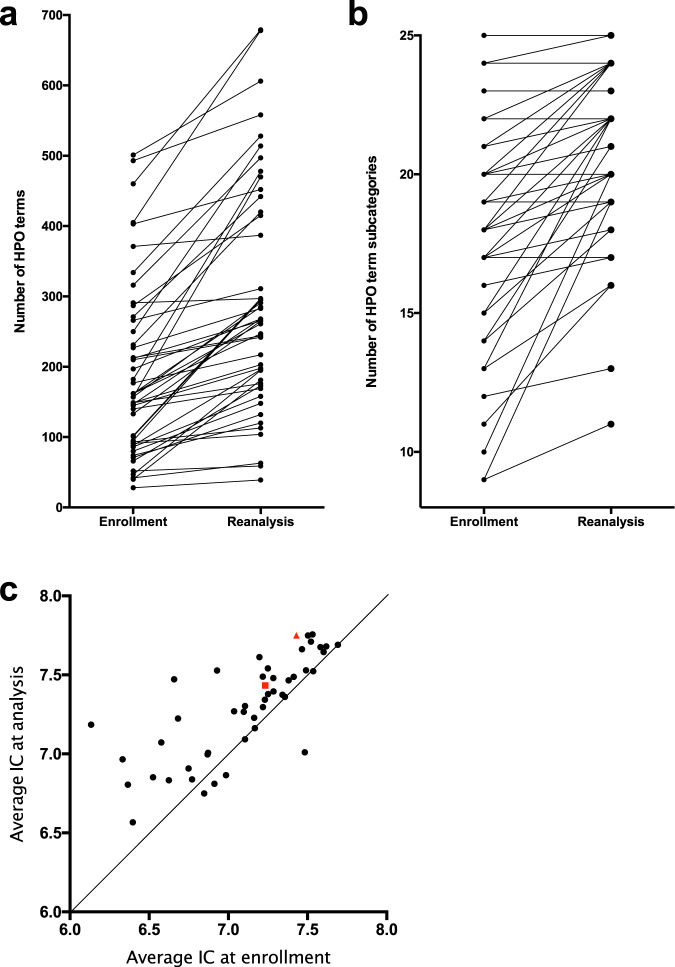
Characterization of HPO term lists automatically extracted from patient EHRs. **a** The number of HPO terms extracted at enrollment and reanalysis, with each case represented by two linked points. **b** The number of HPO subcategories (out of 28 total) represented by terms extracted at enrollment and reanalysis. **c** The average information content (IC) of HPO terms extracted at enrollment and reanalysis differed significantly (Wilcoxon paired signed-rank test; *p* < 0.0001). Each case is represented by one point. The two cases for which diagnoses were reported following reanalysis are represented by a red square (case 6009) and a red triangle (case 6033).

Although CNLP-based phenotyping was not used during the initial analysis, we wished to understand whether this method captures the evolution of patient phenotypes. Therefore, we extracted Human Phenotype Ontology (HPO) terms from patient EHRs delimited by the date of enrollment and compared these with terms extracted at the time of reanalysis. The median number of HPO terms generated by automated phenotyping was 160 at enrollment (range: 28–501) and 267 at reanalysis (range: 39–679), representing a median increase of 79 terms, or 55% (*p* < 0.0001, Wilcoxon paired signed-rank test; Fig. 2a, Supplementary Table 2). HPO terms comprise 28 broad subcategories, most of which correspond to distinct body organ systems. The average number of subcategories represented by HPO terms increased by 2.35 from enrollment to reanalysis (*p* < 0.0001, Wilcoxon paired signed-rank test; Fig. 2b, Supplementary Table 3). Furthermore, the mean information content of the extracted HPO terms (defined as inverse of the logarithm of the probability of a term’s presence in all OMIM disease descriptions) also increased between enrollment and reanalysis (*p* < 0.0001, Wilcoxon paired signed-rank test; Fig. 2c). These results show that patient phenotypes tend to broaden and deepen through time and that CNLP can effectively capture this evolution.

**Fig. 3:**
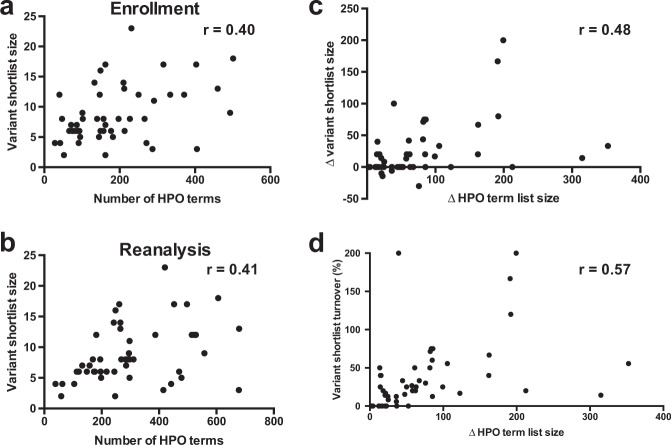
Characteristics of HPO term lists extracted from patient EHRs are correlated with characteristics of resultant variant shortlists. Each case is represented by one point. **a** The number HPO terms extracted at enrollment is positively correlated with the number of Moon variants on the resultant variant shortlist (*r* = 0.40, *p* = 0.005). **b** The number HPO terms extracted at reanalysis is positively correlated with the number of Moon variants on the resultant shortlist (*r* = 0.41, *p* = 0.0032). **c** The change in input HPO term list size between enrollment and reanalysis is positively correlated with the change in resultant variant shortlist size (*r* = 0.48, *p* = 0.0005), as well as with **d** variant shortlist turnover (*r* = 0.57, *p* < 0.0001).

**Fig. 4:**
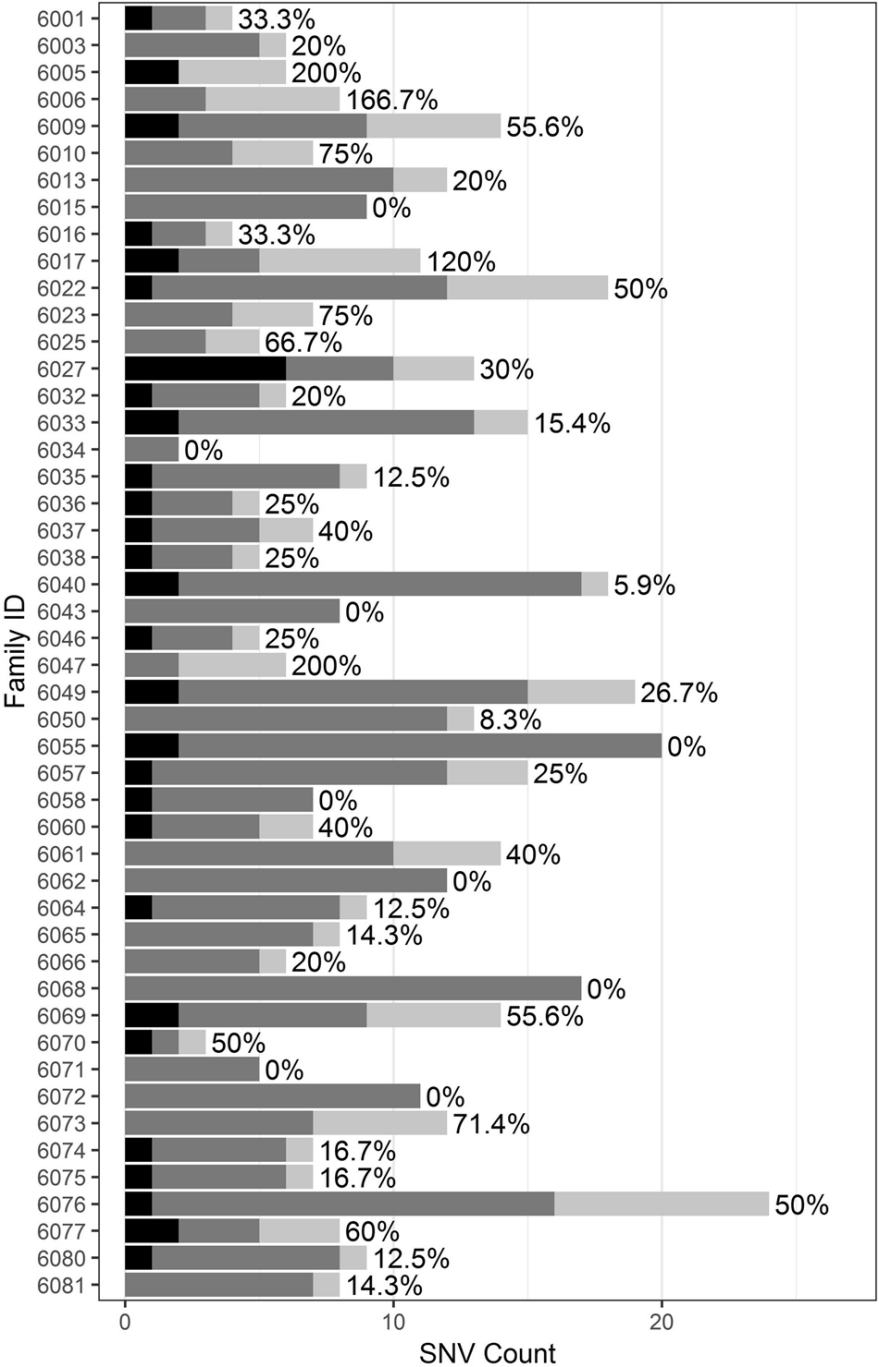
Overlapping variant shortlists at enrollment and reanalysis. Cases are ordered chronologically by date at enrollment. Black: variants on initial enrollment shortlist only; medium gray: variants on enrollment and reanalysis shortlists; light gray: variants on reanalysis shortlist only. The percentage next to each case’s bar reflects the number of new variants on the reanalysis shortlist, relative to the size of the initial enrollment shortlist (median = 25%).

To compare the variant shortlists generated by Moon automated analysis at these two time points, each patient’s VCF was analyzed twice using the same version of Moon (v. 3.0.3) but with either the enrollment or reanalysis HPO lists. The size and content of the variant shortlists output by Moon correlated with the size and content of the input HPO term lists. The median shortlist size increased from 7 variants at enrollment (range: 2–20) to 8 variants at reanalysis (range: 2–23), representing a median increase of 4.2% (*p* < 0.0001; Wilcoxon paired signed-rank test). The variant shortlist size correlated positively with the input HPO list size at both enrollment and reanalysis (Fig. 3a, b; *r* = 0.40, *p* = 0.005 at enrollment; *r* = 0.41, *p* = 0.0032 at reanalysis). Furthermore, the proportional increase in input HPO term list size at reanalysis correlated positively with both the absolute change in resultant variant shortlist size (*r* = 0.48, *p* = 0.0005; Fig. 3c) and with variant “turnover” (i.e., the percentage of new variants present on the reanalysis shortlist relative to the size of the initial shortlist (*r* = 0.57, *p* < 0.0001, Fig. 3d). The initial enrollment and reanalysis variant shortlists overlapped at least partially in all but one case (6005), with a median “turnover” of 25% (Fig. 4).

For the two cases with new diagnoses upon reanalysis, we examined how the patients’ phenotypic evolution was captured in the EHR-extracted HPO terms and subsequent ranking of the diagnostic variant within the shortlist, when run with the same version of Moon (v. 3.0.3). For case 6009, the number of HPO terms generated using automated phenotyping increased from 250 to 514 between enrollment and reanalysis. During variant shortlist generation, each variant was ranked in part based on overlap between the associated gene–disease model and the input HPO terms for that case. For case 6009, the causal *IGF2* variant was ranked #2 at initial enrollment and #3 at reanalysis. Of the input HPO terms, 17/250 (6.8%) at enrollment and 30/514 (5.8%) at reanalysis contributed to ranking of the *IGF2* variant (Supplementary Table 4). For case 6033, the causal *ERCC6* variants were ranked #1 and #2 at both time points. The number of HPO terms increased from 460 to 679 between enrollment and reanalysis, with 54/460 (11.7%) at enrollment and 74/679 (10.9%) at reanalysis contributing to the ranking of the *ERCC6* variants (Supplementary Table 5). For both cases, a small number of phenotypic features characteristic of the diagnosed disorder were present only in the reanalysis HPO term list: for example, developmental delay and short stature in case 6009; and hepatomegaly, splenomegaly, and carious teeth in case 6033. Nevertheless, the ranking of the diagnostic variants did not change substantially for either case.

## Discussion

We present here a partially automated WGS reanalysis pipeline that relies on CNLP of patient EHRs to automate phenotyping and downstream generation of a variant shortlist that has been previously shown to achieve high sensitivity when compared with fully manual curation by experienced analysts^10^. Variant shortlists contained a median of 11 variants, representing a reduction from median of 122 under a manual reanalysis protocol, and offering a solution to mitigate the burden of expert time typically required for reanalysis of NGS data.

Four of the six potential diagnoses identified on reanalysis were not returned to patients on clinical reports, largely due to the corresponding variants’ classification as VUSs. The IRB protocol under which participants were consented for this study allowed only for the reporting of variants classified as Likely Pathogenic or Pathogenic. Future developments, such as reports of these variants in patients with similar phenotypes or functional studies demonstrating their pathogenicity (or lack of pathogenicity) have the potential to alter their classification.

The pipeline presented here differs from those used in other NGS cohort reanalysis studies primarily in its greater incorporation of automation. In contrast to the standard practice of manual assessment and encoding of phenotyping data, it relies on automated phenotyping from the EHR^1–6,12^. Second, while all analysis and reanalysis pipelines rely on automated variant annotation and filtering on variables such as sequence quality and population allele frequencies, this pipeline generates a stringently filtered variant list also accounting for the phenotypic terms input for the patient. This shortlist represents a dramatic reduction in the total number of variants to be considered by the analyst, allowing manual interpretation to proceed more quickly, or to be undertaken by individuals lacking the advanced training needed to correctly filter and evaluate hundreds or thousands of variants^1^.

Using the partially automated reanalysis pipeline described here, we report a yield of 4.2% (2/48, 95% confidence interval (CI): 0.5–14.3%), which is comparable to but slightly lower than other reported NGS reanalysis yields^1–6,12^. This study differs from previously reported reanalysis efforts in several ways that may contribute to the slightly lower yield that we report. First, the analysis was predominantly performed on newborns and infants. The 48 negative cases eligible for reanalysis had a median age of 4.8 months at enrollment and 25 months at reanalysis. Other reanalysis cohorts have median ages ranging from 4 to 6.7 years at enrollment^1–3,5,6,12–14^. Many genetic syndromes are not easily recognizable at early ages, potentially complicating diagnosis even at the reanalysis time point used in this study. Second, our inclusion criteria were intentionally broad, requiring not that patients have a suspected genetic disorder but rather that their phenotypic features be potentially attributable to a genetic disorder. In addition, no major technical changes that are typically included in the reanalysis literature (such as sequencing improvements or bioinformatics pipeline upgrades) were included in this study. Use of the original VCF file generated at the time of analysis, for reanalysis, represents a potential source of data processing savings that may be attractive to clinical laboratories wishing to implement iterative reanalysis.

Although new gene–disease discovery is a major factor driving diagnosis upon reanalysis, all six diagnoses (reported and possible) in this reanalysis cohort were variants found in genes known to be involved in human disease at the time of initial analysis^1^. In the intervening time between analysis and reanalysis, these diagnoses became more compelling due to several factors, including variant publication or classification in ClinVar in connection with disease, or functional testing of patient cells that provided orthogonal support to a diagnosis (6033). In case 6009, the diagnosis was initially missed due to a manual error in data labeling, which was discovered only upon reanalysis. Thus, in addition to the benefit of greater efficiency, a partially automated reanalysis pipeline such as the one described here can serve a quality control function, flagging errors made in patient data processing during analysis.

The number of HPO terms extracted from patient EHRs via automated phenotyping increased from a median of 160 to 267 terms between enrollment and reanalysis, and this increase correlated with the “turnover” in the variants included on the variant shortlist. While both diagnostic variants reported from this reanalysis cohort were highly ranked in the shortlist using HPO terms from the time of enrollment as well as reanalysis, future cases are likely to benefit from the sensitivity of the shortlist algorithm to new phenotypic input.

As genomic data and knowledge of genetic disorders continue to accumulate rapidly, reanalysis of initially negative cases will likely become a standard practice for many clinical laboratories. Although many clinical laboratories provide some mechanism for NGS reanalysis, either by provider request or through an internal procedure, these efforts are limited by the strain on staffing resources posed by reanalysis. Partial automation may ease this burden, allowing more regular reanalysis. Outside of the scope of this publication, but of importance in the realm of NGS reanalysis, are the questions of how to initially prepare patients or research subjects for the possibility of a reanalysis result delivery years after initial testing and how to responsibly deliver NGS reanalysis results^15^. Ideally, reanalysis should be repeated periodically for all cases that remain negative and should incorporate new clinical information for the patient^16^. The four cases with possible (but not reportable) diagnoses presented here illustrate the uncertainty that can remain in clinical NGS cases for which a negative report has been issued and the benefit of performing iterative reanalysis. Future studies may examine the utility of such iterative analysis using this automated pipeline.

## Methods

### Study design

Retrospective comparison of the diagnostic utility of reanalysis of WGS by manual and partially automated methods was approved by the IRB at the University of California, San Diego (UCSD; Project #160468). Inpatients at RCHSD without etiologic diagnoses, in whom a genetic disorder was possible, were nominated for diagnostic, rapid WGS by diverse clinicians from 26 July 2016 to 3 April 2017. Informed consent was obtained from at least one parent or guardian of each patient included in the study. Of the 82 children who received rapid WGS during this period, 48 who received WGS that was not diagnostic at initial manual analysis and for whom at least 1 year had elapsed since initial analysis were studied herein. The clinical characteristics of 26 of the 48 children have been previously reported (Supplementary Table 2)^17,18^.

### WGS and manual analysis/reanalysis

WGS was performed on DNA extracted from the blood samples of study participants as previously described^17,18^. Briefly, DNA was sequenced with Illumina (San Diego, CA) HiSeq 2500 or 4000 instruments with paired 101-nt reads. Alignment and nucleotide variant calling was performed using the DRAGEN hardware and software platform (version 2.1.5)^10^. Yield ranged from 115.8 to 239.8 Gb, resulting in 4,765,952 to 5,654,509 variant calls per individual and an average of 45.3× coverage. Analysis considered single-nucleotide variants (SNVs) and small insertions and deletions only.

VCF files from DRAGEN were annotated and analyzed in Opal Clinical versions 4.20–4.28 (Fabric Genomics, Oakland, CA) according to standard guidelines^19,20^. Manual variant analysis relied on a number of tools and resources, including variant ranking tools Phevor and VAAST, population frequency databases such as ExAC and gnomAD, in silico damage prediction scores (including SIFT, MutationTaster, and Polyphen), the Human Gene Mutation Database, ClinVar, literature searches, and manual inspection of reads using the Integrative Genomics Viewer. Manual reanalysis variant counts were generated using the same tools and resources, following the current variant filtering protocols used by Rady Children’s Institute for Clinical Genomics for manual analysis/reanalysis. Tool version details are as follows: VAAST: 1.1; dbSNP: 147–149; Genome Reference Consortium Human Genome Build v37; ExAC: 0.3; SIFT, MutationTaster, PolyPhen: dbNSFP v.2.9; HGMD: 2017.1–20.17.2; ClinVar: May 26, 2016-March 27, 2017 weekly releases; IGV: 2.3.76–2.3.86. Phenotypic features were manually extracted from EHRs by analysts, and interpretation was performed on trios in 28 families, duos in 11 families, and the proband only in 7 families. CNV calls, which were analyzed separately from analysis and reanalysis, were generated after realignment and variant calling with DRAGEN 3.4.5, using an automated pipeline that integrates the tools Manta and CNVnator as previously described^21,22^.

### Partially automated reanalysis

HPO terms were re-extracted from patient records at the time of reanalysis, using CLiX ENRICH (Clinithink, Alpharetta, GA) as previously described^10^. Briefly, unstructured clinical records were transformed into JSON format, encoded as SNOMED CT expressions by CLiX ENRICH, and transformed to an HPO list using a CLiX query map. Study participant VCF files, together with HPO term lists from CLiX ENRICH, were uploaded to Moon (Diploid, Leuven, Belgium) (Version details: ClinVar: 2018-04-29; dbNSFP: 3.5; dbSNP: 150; dbscSNV: 1.1; Apollo: 2018-05-03; Ensembl: 37; gnomAD: 2.0.1; HPO: 2017-10-05; Moon: 2.0.3) for automated, phenotype-driven variant analysis, as previously described^10^. For comparisons of Moon variant shortlists with HPO terms drawn from EHRs at enrollment or reanalysis, a newer version of Moon (Version details: ClinVar: 2019-03-11; dbNSFP: 3.5; dbSNP: 150; dbscSNV: 1.1; Apollo: 2019-03-18; Ensembl: 37; gnomAD: 2.0.1; HPO: 2019-02-12; KB: 2019-04-03; DGV: 2016-03-01; dbVar: 2018-06-24; Mitomap: 2019-01-14; Mitimpact: 2.9.1; Mastermind: 2018-11-26; Moon: 3.0.3) was used.

### CNV analysis

Copy number analysis was performed as previously described^23^. Briefly, the read pair-based tool, Manta, was used to detect smaller CNVs while the coverage-based caller, CNVnator, was used to detect larger CNVs. Calls were filtered for events overlapping known disease genes and filtered by an internal allele frequency of <2%.

### Statistical analysis

Nonparametric Spearman correlations, Wilcoxon signed-rank tests, and corresponding two-tailed *p* values were calculated using Prism (version 6.0h, GraphPad, La Jolla, CA). The 95% CI for the proportion of new diagnoses made upon reanalysis was calculated using the binomial exact (Clopper–Pearson) method. The information content of each HPO term was calculated as previously described^10^.

### Reporting summary

Further information on research design is available in the Nature Research Reporting Summary linked to this article.

## Supplementary information

Supplementary Information

Reporting Summary

## Data Availability

The variant list data that were used in reanalysis are available as Supplementary Information. The sequencing data that the variant lists are drawn from are available from the corresponding author upon reasonable request and completion of a data use agreement, subject to the limitations of the informed consent documents for each subject.
