# Supplementary material for: Partially automated whole-genome sequencing reanalysis of previously undiagnosed pediatric patients can efficiently yield new diagnoses
Source: NPJ Genom Med. 2020 Aug 11;5:33. doi: 10.1038/s41525-020-00140-1 (PMC7419288; doi:10.1038/s41525-020-00140-1)
Supplement: Supplementary file 1 — Supplementary Information [file 41525_2020_140_MOESM1_ESM.pdf]

Supplementary Figures and Tables

Supplementary Table 1: Variant shortlist contents Variant details and annotations provided in Moon shortlists and used for pathogenicity assessment for the 48 cases

Case	Chr	Pos	Ref	Alt	Genotype	Gene	Disorder	HGVS_C notation	HGVS_P notation	gnomAD frequency	Allele Depth	Genotype Quality	SIFT	POLYPHEN	MUT TASTER
6001	17	29508776	T	C	T/C	NF1	Neurofibromatosis, type I	c.703T>C	p.Tyr235His	8.13E-06	40,30	99	0.521	0.007	1
6001	14	30135303	C	T	C/T	PRKD1	Congenital heart defects and ectodermal dysplasia	c.515G>A	p.Arg172His	1.63E-05	24,27	99	0	0.997	1
6001	21	45712241	G	A	G/A	AIRE	Autoimmune polyendocrine syndrome, type i, with or without reversible metaphyseal dysplasia	c.1052G>A	p.Arg351Gln	2.36E-05	29,30	99	0.896	0.006	1
6001	2	208631753	C	G	C/G	FZD5	Coloboma, non-syndromic, FZD5-related	c.1711G>C	p.Gly571Arg	7.87E-06	21,26	99	0.065	0	0.99
6001	12	133250174	G	A	G/A	POLE	Colorectal cancer, susceptibility to, 12	c.1346C>T	p.Thr449Met	3.27E-05	28,23	99	0.092	0.028	1
6001	14	105173896	CTGGTTCCAGTGCCGAG	C	CTGGTTCCAGTGCCGAG/C	INF2	Charcot-marie-tooth disease, dominant intermediate e	c.1293_1308delTGGTTCCAGTGCCGAG	p.Gly432fs	1.61E-05	13,4	99	n/a	n/a	n/a
6003	2	166900340	A	T	A/T	SCN1A	Epileptic encephalopathy, early infantile, 6	c.1882T>A	p.Ser628Thr	1.22E-05	33,28	99	0.085	0.005	0.611
6003	3	189612188	G	A	G/A	TP63	Split-hand/foot malformation 4	c.1940G>A	p.Arg647His	0	28,19	99	0.001	1	1
6003	19	1621885	C	T	C/T	TCF3	Agammaglobulinemia 8, autosomal dominant	c.754G>A	p.Gly252Ser	1.97E-05	19,21	99	1	0.064	1
6003	9	131452285	A	T	A/T	SET	Intellectual disability, SET-related	c.20A>T	p.Gln7Leu	0	13,18	99	0.09	0.069	1
6003	1	1451415	C	G	C/G	ATAD3A	Harel-Yoon syndrome	c.229C>G	p.Leu77Val	4.34867E-04	29,25	99	0.121	0.841	1
6003	19	15272414	G	A	G/A	NOTCH3	Cerebral arteriopathy, autosomal dominant, with subcortical infarcts and leukoencephalopathy, type 1	c.6025C>T	p.Arg2009Trp	4.17E-05	21,31	99	0	1	1
6005	12	7177488	C	T	C/T	C1S	Ehlers-danlos syndrome, periodontal type, 2	c.1600C>T	p.Arg534Trp	0.00014234	19,33	99	0.002	0.867	0
6005	7	103216122	G	T	G/T	RELN	Epilepsy, familial temporal lobe, 7	c.4176C>A	p.Ser1392Arg	1.63E-05	25,29	99	0.268	0.952	1
6005	3	63975913	G	C	G/C	ATXN7	Spinocerebellar ataxia 7	c.1423G>C	p.Glu475Gln	0	23,23	99	0.062	0.994	1
6005	19	42777790	G	A	G/A	CIC	Mental retardation, autosomal dominant 45	c.1855G>A	p.Val619Ile	3.25E-05	19,15	99	n/a	n/a	0.984
6005	22	24911329	G	A	G/A	UPB1	Beta-ureidopropionase deficiency	c.782G>A	p.Gly261Glu	7.72E-05	16,23	99	0.074	0.318	1
6005	22	24906800	T	G	T/G	UPB1	Beta-ureidopropionase deficiency	c.448T>G	p.Phe150Val	7.31E-05	35,31	99	0.15	0.191	1

6005	2	238249550	G	A	G/A	COL6A3	Ullrich congenital muscular dystrophy 1	c.8009C>T	p.Ala2670Val	6.19135E-04	20,13	99	0.008	0.699	0.999
6005	14	105173903	CAGTG	C	CAGTG/C	INF2	Charcot-marie-tooth disease, dominant intermediate e	c.1300_1303del AGTG	p.Ser434fs	0	21,6	99	n/a	n/a	n/a
6005	14	105173896	CTGGT T	C	CTGGTT/ C	INF2	Charcot-marie-tooth disease, dominant intermediate e	c.1293_1297del TGGTT	p.Gly432fs	1.61E-05	20,8	99	n/a	n/a	n/a
6006	14	105173896	CTGGT TCCAG TGCCG AG	C	CTGGTTC CAGTGCC GAG/C	INF2	Charcot-marie-tooth disease, dominant intermediate e	c.1293_1308del TGGTTCCAGTGC CGAG	p.Gly432fs	1.61E-05	9,5	99	n/a	n/a	n/a
6006	18	42530552	A	G	A/G	SETBP1	Schinzel-giedion midface retraction syndrome	c.1247A>G	p.His416Arg	4.08E-06	23,40	99	0.003	0.812	0.941
6006	16	11647515	G	A	G/A	LITAF	Charcot-marie-tooth disease, demyelinating, type 1c	c.251C>T	p.Pro84Leu	1.22E-05	17,27	99	0	0.934	1
6006	20	57876579	AGACT GTGG CTGGC CCTGG CGAG GG	A	AGACTGT GGCTGGC CCTGGCG AGGG/A	EDN3	Central hypoventilation syndrome, congenital	c.177_200delTG GCCCTGGCGAG GGGACTGTGGC	p.Gly60_Ala6 7del	2.46E-05	17,26	99	n/a	n/a	n/a
6006	2	25990472	C	T	C/T	ASXL2	Shashi-Pena syndrome	c.755G>A	p.Arg252Lys	8.13E-06	37,25	99	0.029	0.943	1
6006	14	91744372	G	C	G/C	CCDC88C	Hydrocephalus, nonsyndromic, autosomal recessive 1	c.4952C>G	p.Thr1651Arg	0	16,27	99	0.473	0.001	1
6006	14	91739753	G	C	G/C	CCDC88C	Hydrocephalus, nonsyndromic, autosomal recessive 1	c.5303C>G	p.Pro1768Arg	0	23,22	99	0.1	0.045	1
6006	12	122255296	C	T	C/T	SETD1B	Intellectual disability, epilepsy and autism, SETD1B-related	c.2998C>T	p.Arg1000Trp	7.55E-06	21,27	99	0	0.998	0.999
6006	4	55127534	G	A	G/A	PDGFRA	Gastrointestinal stromal tumor	c.322G>A	p.Glu108Lys	1.22E-05	24,30	99	0.191	0.045	1
6009	11	2156655	G	T	G/T	IGF2	Growth restriction, severe, with distinctive facies	c.267C>A	p.Cys89*	0	21,27	99	n/a	n/a	1
6009	17	7350875	C	G	C/G	CHRNB1	Myasthenic syndrome, congenital, 2A, slow-channel	c.516C>G	p.Tyr172*	4.47E-05	31,19	99	n/a	n/a	1
6009	7	42006221	GTGT	G	GTGT/G	GLI3	Pallister-hall syndrome	c.2447_2449del ACA	p.Asn816del	4.06E-06	20,23	99	n/a	n/a	n/a
6009	11	17793728	A	G	A/G	KCNC1	Epilepsy, progressive myoclonic 7	c.1087A>G	p.Ile363Val	0	20,17	99	0.607	0.22	1
6009	7	94037529	T	C	T/C	COL1A2	Osteogenesis imperfecta, type ii	c.674T>C	p.Val225Ala	0	37,26	99	0.901	0.004	0.997
6009	3	47162805	C	A	C/A	SETD2	Luscan-Lumish syndrome	c.3321G>T	p.Glu1107Asp	0	31,28	99	0.089	0.066	0.916
6009	2	25966820	C	T	C/T	ASXL2	Shashi-Pena syndrome	c.2386G>A	p.Ala796Thr	1.62E-05	27,27	99	0.002	0.007	0.893
6009	19	40904651	C	A	C/A	PRX	Hypertrophic neuropathy of dejerine-sottas	c.257G>T	p.Cys86Phe	1.23E-05	19,32	99	0.111	0.994	1

6009	19	39214972	G	T	G/T	ACTN4	Focal segmental glomerulosclerosis 1	c.1868G>T	p.Trp623Leu	1.63E-05	26,27	99	0.002	0.999	1
6009	18	28648975	C	A	C/A	DSC2	Arrhythmogenic right ventricular dysplasia, familial, 11	c.2393G>T	p.Arg798Leu	4.47E-05	32,23	99	0.008	0.237	1
6010	12	49424779	T	C	T/C	KMT2D	Kabuki syndrome 1	c.13568A>G	p.Lys4523Arg	4.06E-06	23,15	99	0.004	0.994	1
6010	17	78155440	T	C	T/C	CARD14	Psoriasis 2	c.203T>C	p.Met68Thr	4.96E-05	21,23	99	0	0.991	1
6010	12	21970150	A	G	A/G	ABCC9	Cantu syndrome	c.3863T>C	p.Met1288Thr	4.07E-06	23,30	99	0.872	0.001	0.993
6010	10	88476180	CTG	C	CTG/C	LDB3	Cardiomyopathy, dilated, 1c, with or without left ventricular noncompaction	c.1344_1345delTG	p.Ala449fs	3.70E-05	16,6	99	n/a	n/a	n/a
6010	10	88476194	T	C	T/C	LDB3	Cardiomyopathy, dilated, 1c, with or without left ventricular noncompaction	c.1357T>C	p.Ser453Pro	2.51E-05	15,4	99	0.071	0.003	0.993
6013	10	76603040	C	T	C/T	KAT6B	Genitopatellar syndrome	c.425C>T	p.Thr142Ile	4.07E-06	20,24	99	0.448	0.001	0.995
6013	11	64032494	CA	C	CA/C	PLCB3	Spondylometaphyseal dysplasia with corneal dystrophy, PLCB3-related	c.2725delA	p.Thr909fs	1.70E-05	11,7	99	n/a	n/a	n/a
6013	20	9438093	CAG	C	CAG/C	PLCB4	Auriculocondylar syndrome 2	c.2995_2996delGA	p.Glu999fs	2.04E-05	23,28	99	n/a	n/a	n/a
6013	3	143185993	T	C	T/C	SLC9A9	Autism, susceptibility to, 16	c.1355A>G	p.Asn452Ser	2.03E-05	31,23	99	0.04	0.029	1
6013	2	241710458	G	A	G/A	KIF1A	Mental retardation, autosomal dominant 9	c.1271C>T	p.Ala424Val	0	17,22	99	0.46	0.026	1
6013	2	238274544	C	T	C/T	COL6A3	Ullrich congenital muscular dystrophy 1	c.5635G>A	p.Gly1879Ser	8.13E-05	25,14	99	0.277	0.41	1
6013	2	179398629	C	T	C/T	TTN	Hereditary myopathy with early respiratory failure	c.102713G>A	p.Arg34238His	2.03E-05	28,17	99	0.025	0.353	0.825
6013	11	64032499	CCAG	C	CCAG/C	PLCB3	Spondylometaphyseal dysplasia with corneal dystrophy, PLCB3-related	c.2731_2733delAGC	p.Ser911del	2.82997E-04	12,6	99	n/a	n/a	n/a
6013	16	90000079	G	T	G/T	TUBB3	Cortical dysplasia, complex, with other brain malformations 1	c.370G>T	p.Val124Phe	1.26E-05	31,33	99	0	n/a	1
6013	2	70441626	G	C	G/C	TIA1	Welander distal myopathy	c.889C>G	p.Gln297Glu	2.52E-05	23,23	99	0.226	0.023	0.999
6013	11	64032507	CTGGATGCCT	C	CTGGATGCCT/C	PLCB3	Spondylometaphyseal dysplasia with corneal dystrophy, PLCB3-related	c.2738_2746delTGGATGCCT	p.Leu913_Ser916delinsPro	0	12,4	99	n/a	n/a	n/a
6013	22	46202953	G	A	G/A	ATXN10	Spinocerebellar ataxia 10	c.385G>A	p.Ala129Thr	4.06E-06	26,25	99	n/a	n/a	n/a
6013	18	31325896	CTTGGCTTTG	C	CTTGGCTTTG/C	ASXL3	Bainbridge-Ropers syndrome	c.6085_6093delTTGGCTTTG	p.Leu2029_Leu2031del	4.32E-05	26,11	99	n/a	n/a	n/a
6015	17	72869069	T	G	T/G	FDXR	Mitochondriopathy and optic atrophy, FDXR-related	c.1A>C	p.Met1?	2.06E-05	29,34	99	0.159	0.004	1
6015	17	72862928	G	A	G/A	FDXR	Mitochondriopathy and optic atrophy, FDXR-related	c.377C>T	p.Ala126Val	8.13E-06	25,39	99	0	0.951	1
6015	2	29541235	C	T	C/T	ALK	Neuroblastoma, susceptibility to, 3	c.1582G>A	p.Ala528Thr	3.66E-05	37,22	99	0.475	0.001	1

6015	12	103352162	C	CA	C/CA	ASCL1	Central hypoventilation syndrome, congenital	c.140_141insA	p.Gln48fs	3.16E-05	21,17	99	n/a	n/a	n/a
6015	12	103352157	G	GC	G/GC	ASCL1	Central hypoventilation syndrome, congenital	c.135_136insC	p.Ala46fs	2.42E-05	20,17	99	n/a	n/a	n/a
6015	12	103352146	GCCGCCGC	G	GCCGCCGC/G	ASCL1	Central hypoventilation syndrome, congenital	c.125_131delCCGCCGC	p.Ala42fs	3.34E-05	21,18	99	n/a	n/a	n/a
6015	12	103352140	GC	G	GC/G	ASCL1	Central hypoventilation syndrome, congenital	c.120delC	p.Ala41fs	1.78E-05	22,16	99	n/a	n/a	n/a
6015	11	47361308	C	T	C/T	MYBPC3	Cardiomyopathy, familial hypertrophic, 4	c.1961G>A	p.Arg654His	2.44E-05	32,23	99	0.402	0.007	0.819
6015	X	13773347	G	T	T	OFD1	Orofaciodigital syndrome I	c.1207G>T	p.Val403Leu	0	0,32	96	0.126	0.015	1
6015	9	98242260	T	C	T/C	PTCH1	Basal cell nevus syndrome	c.1058A>G	p.Lys353Arg	1.62E-05	30,30	99	0.392	0.025	0.744
6015	6	117997008	C	T	C/T	NUS1	Mental retardation, autosomal dominant 55, with seizures	c.175C>T	p.Pro59Ser	7.86E-06	18,16	99	0.671	0.001	0.988
6015	2	179498202	T	G	T/G	TTN	Salih myopathy	c.42884A>C	p.Asp14295Ala	0	28,33	99	0.005	0.999	1
6015	2	179495039	C	A	C/A	TTN	Salih myopathy	c.44210G>T	p.Arg14737Leu	2.20112E-04	24,25	99	0.226	0.005	0.999
6015	19	50099382	C	T	C/T	PRR12	Intellectual disability and iris abnormalities, PRR12-related	c.1790C>T	p.Ala597Val	2.15E-05	34,27	99	0.08	0.014	1
6015	12	103352165	AGAGCGC	A	AGAGCGC/A	ASCL1	Central hypoventilation syndrome, congenital	c.145_150delAGCGCG	p.Ser49_Ala50del	3.20E-05	22,18	99	n/a	n/a	n/a
6015	12	103352130	AGCC	A	AGCC/A	ASCL1	Central hypoventilation syndrome, congenital	c.111_113delCGC	p.Ala38del	1.61E-05	21,16	99	n/a	n/a	n/a
6015	12	39688239	A	C	A/C	KIF21A	Fibrosis of extraocular muscles, congenital, 1/3B	c.5015T>G	p.Ile1672Ser	1.63E-05	33,30	99	0.007	0.001	1
6015	11	78412820	G	T	G/T	TENM4	Tremor, hereditary essential, 5	c.4838C>A	p.Thr1613Asn	0	31,28	99	0.346	0.081	0.996
6015	5	14507325	G	C	G/C	TRIO	Mental retardation, autosomal dominant 44	c.8707G>C	p.Val2903Leu	0	18,26	99	1	0.125	1
6015	2	21232758	A	C	A/C	APOB	Hypobetalipoproteinemia, familial, 1	c.6982T>G	p.Phe2328Val	0	29,20	99	0.002	0.038	0.556
6015	14	105173896	CTGGTTCCAGTGCCGAG	C	CTGGTTCCAGTGCCGAG/C	INF2	Charcot-marie-tooth disease, dominant intermediate e	c.1293_1308delTGGTTCCAGTGCCGAG	p.Gly432fs	1.61E-05	19,5	99	n/a	n/a	n/a
6015	4	113566766	T	C	T/C	LARP7	Alazami syndrome	c.262T>C	p.Ser88Pro	1.18083E-04	25,7	99	n/a	n/a	n/a
6015	4	113566757	T	C	T/C	LARP7	Alazami syndrome	c.253T>C	p.Ser85Pro	4.09103E-04	24,8	99	n/a	n/a	n/a
6015	18	72997858	A	C	A/C	TSHZ1	Aural atresia, congenital	c.496A>C	p.Ser166Arg	1.53E-05	14,4	99	0.016	0.08	1
6015	18	72997856	T	C	T/C	TSHZ1	Aural atresia, congenital	c.494T>C	p.Val165Ala	8.26E-06	13,5	99	1	0	1
6016	1	196744079	G	A	G/A	CFHR3	Hemolytic uremic syndrome, atypical, susceptibility to, 1	c.58+5G>A		4.29E-06	23,11	99	n/a	n/a	n/a
6016	11	58892376	C	CA	C/CA	FAM111B	Poikiloderma, hereditary fibrosing, with tendon contractures, myopathy,	c.816dupA	p.Ala273fs	8.40E-06	24,16	99	n/a	n/a	n/a

							and pulmonary fibrosis							
6016	12	2224519	G	A	G/A	CACNA1C	Timothy syndrome	c.179G>A	p.Arg60Gln	4.87E-06	25,29	99	0	0.62	1
6016	8	144995200	G	A	G/A	PLEC	Epidermolysis bullosa simplex, ogna type	c.9200C>T	p.Ser3067Leu	3.67E-05	19,27	99	0.002	0.886	1
6016	12	6441061	G	A	G/A	TNFRSF1A	Periodic fever, familial, autosomal dominant	c.650C>T	p.Pro217Leu	3.28E-05	30,18	99	0	0.713	1
6016	1	45292392	C	T	C/T	PTCH2	Basal cell nevus syndrome	c.2744G>A	p.Arg915His	4.31E-06	26,23	99	0.755	0.001	1
6016	1	231124117	A	G	A/G	ARV1	Epileptic encephalopathy, early infantile, 38	c.184A>G	p.Ile62Val	2.88E-05	24,24	99	0.422	0.028	1
6016	1	231114898	T	G	T/G	ARV1	Epileptic encephalopathy, early infantile, 38	c.5T>G	p.Val2Gly	4.13E-06	19,20	99	0.012	0.007	1
6016	7	151372550	T	C	T/C	PRKAG2	Glycogen storage disease of heart, lethal congenital	c.640A>G	p.Arg214Gly	8.13E-06	25,29	99	0.039	0.016	0.998
6016	X	49082418	A	C	A/C	CACNA1F	Aland island eye disease	c.1637T>G	p.Ile546Ser	0	34,22	99	0.042	0.013	0.902
6016	22	36691584	G	A	G/A	MYH9	Epstein syndrome	c.3452C>T	p.Thr1151Met	1.22E-05	25,24	99	0.001	0.99	1
6016	15	57484474	A	G	A/G	TCF12	Craniosynostosis 3	c.509A>G	p.His170Arg	0	26,18	99	0.095	0.535	0.75
6016	14	30133009	C	T	C/T	PRKD1	Congenital heart defects and ectodermal dysplasia	c.592G>A	p.Gly198Ser	4.06E-06	27,24	99	0	0.378	1
6016	1	244006457	T	C	T/C	AKT3	Megalencephaly-polymicrogyria-polydactyly-hydrocephalus syndrome 2	c.16A>G	p.Ile6Val	4.06E-05	25,32	99	0.75	0.007	0.952
6016	6	52288790	A	G	A/G	EFHC1	Epilepsy, myoclonic juvenile	c.110A>G	p.Tyr37Cys	0	18,24	99	0.003	0.992	0.993
6016	21	27484391	G	A	G/A	APP	Alzheimer disease	c.130C>T	p.His44Tyr	8.12E-06	22,22	99	0	0.998	1
6016	17	42334902	G	C	G/C	SLC4A1	Renal tubular acidosis, distal, autosomal dominant	c.1442C>G	p.Thr481Ser	4.88E-05	16,25	99	1	0	1
6016	1	161146832	G	C	G/C	PPOX	Porphyria variegata	c.431G>C	p.Gly144Ala	7.71E-06	17,41	99	0	0.001	1
6017	14	105173896	CTGGTTCCAGTGCCGAG	C	CTGGTTCCAGTGCCGAG/C	INF2	Focal segmental glomerulosclerosis 5	c.1293_1308delTGGTTCCAGTGCCGAG	p.Gly432fs	1.61E-05	18,8	99			
6017	X	619504	CT	C,CTT	CTT	SHOX	Leri-weill dyschondrosteosis	c.634-3dupT		0	2,13,12	99			
6017	16	5134829	C	T	C/T	ALG1	Congenital disorder of glycosylation, type ik	c.1342C>T	p.Arg448*	4.39E-05	31,21	99	n/a	n/a	1
6017	2	145274024	C	T	C/T	ZEB2	Mowat-wilson syndrome	n.304+1G>A		0	12,6	99	n/a	n/a	n/a
6017	1	120468135	C	T	C/T	NOTCH2	Hajdu-Cheney syndrome	c.4304G>A	p.Arg1435Gln	2.44E-05	25,26	99	0.04	0.825	0.809
6017	17	42461931	C	G	C/G	ITGA2B	Bleeding disorder, platelet-type, 16	c.822G>C	p.Glu274Asp	4.47E-05	27,28	99	0.313	0.174	0.986
6017	19	15291840	C	T	C/T	NOTCH3	Lateral meningocele syndrome	c.2926G>A	p.Val976Ile	1.84E-05	34,35	99	0.15	0.001	1
6017	20	62328336	G	A	G/A	RTEL1	Dyskeratosis congenita, autosomal recessive 5	c.4123G>A	p.Val1375Met	6.44E-05	17,30	99	n/a	n/a	n/a
6017	7	42012195	G	A	G/A	GLI3	Pallister-hall syndrome	c.1844C>T	p.Thr615Ile	1.24E-05	29,26	99	0.003	0.901	1

6017	20	61050154	A	G	A/G	GATA5	Congenital heart defects, multiple types, 5	c.424T>C	p.Tyr142His	5.94354E-04	25,21	99	0	0.998	1
6017	16	5129091	G	T	G/T	ALG1	Congenital disorder of glycosylation, type ik	c.889G>T	p.Ala297Ser	0	16,36	99	0	0.009	0.999
6017	18	42643437	T	C	T/C	SETBP1	Schinzel-giedion midface retraction syndrome	c.4565T>C	p.Leu1522Pro	0	19,10	99	0.093	0.011	1
6017	12	6094250	G	A	G/A	VWF	Von willebrand disease, type 2	c.6937C>T	p.Arg2313Cys	0.00094617	27,24	99	0.024	0.828	0.785
6017	1	45295333	C	G	C/G	PTCH2	Basal cell nevus syndrome	c.1036G>C	p.Glu346Gln	3.25E-05	21,22	99	0.086	0.452	0.999
6017	1	45293814	C	T	C/T	PTCH2	Basal cell nevus syndrome	c.1759G>A	p.Gly587Arg	4.49E-05	22,17	99	0.596	0.002	1
6017	12	133242017	G	A	G/A	POLE	Colorectal cancer, susceptibility to, 12	c.2339C>T	p.Ser780Leu	2.46E-05	23,29	99	0.009	0.375	1
6022	5	14497073	G	A	G/A	TRIO	Mental retardation, autosomal dominant 44	c.7966G>A	p.Glu2656Lys	1.22E-05	28,25	99	0.019	0.621	1
6022	17	76993500	G	A	G/A	CANT1	Desbuquois dysplasia 1	c.205C>T	p.Pro69Ser	3.34E-05	22,31	99	0.44	0	0.895
6022	17	76989713	C	A	C/A	CANT1	Desbuquois dysplasia 1	c.1125G>T	p.Met375Ile	2.03E-05	20,19	99	0.297	0.083	0.996
6022	1	7886557	T	G	T/G	PER3	Advanced sleep phase syndrome, familial, 3	c.1975T>G	p.Phe659Val	0	35,31	99	0	0	1
6022	10	43606815	G	A	G/A	RET	Central hypoventilation syndrome, congenital	c.1424G>A	p.Arg475Gln	1.22E-05	23,20	99	0.052	0.004	0.997
6022	8	133142093	C	T	C/T	KCNQ3	KCNQ3-related developmental disability	c.2035G>A	p.Asp679Asn	1.62E-05	19,22	99	0.52	0.075	0.999
6022	17	65908825	G	T	G/T	BPTF	Neurodevelopmental disorder with dysmorphic facies and distal limb anomalies	c.5203G>T	p.Asp1735Tyr	2.05E-05	35,30	99	0	0.919	0.67
6022	8	38314997	C	T	C/T	FGFR1	Osteoglophonic dysplasia	c.67G>A	p.Ala23Thr	2.46E-05	17,25	99	1	0	1
6022	14	50655244	G	T	G/T	SOS2	Noonan syndrome 9	c.685C>A	p.Leu229Ile	0	20,31	99	0.333	0.021	0.998
6022	20	60887713	C	T	C/T	LAMA5	Extracellular matrix syndrome, LAMA5-related	c.9202G>A	p.Val3068Met	2.62E-05	22,24	99	0.006	0.793	0.996
6022	9	109687188	A	G	A/G	ZNF462	Craniofacial anomalies, corpus callosum dysgenesis, ptosis, and developmental delay	c.995A>G	p.Asn332Ser	4.06E-06	27,31	99	0.342	0.006	0.823
6022	6	49578786	C	T	C/T	RHAG	Overhydrated hereditary stomatocytosis	c.1018G>A	p.Val340Met	1.63E-05	25,35	99	0.04	0.048	1
6023	19	39016117	C	T	C/T	RYR1	Central core disease of muscle	c.10601C>T	p.Thr3534Met	3.25E-05	19,23	99	0.121	0.01	1
6023	9	75420410	A	T	A/T	TMC1	Deafness, autosomal recessive 7	c.1679A>T	p.Asp560Val	1.62E-05	25,22	99	0	1	1
6023	4	102092350	C	G	C/G	PPP3CA	Epileptic encephalopathy, infantile or early childhood	c.47G>C	p.Arg16Thr	8.11E-06	32,17	99	n/a	n/a	n/a
6023	8	144997670	C	T	C/T	PLEC	Epidermolysis bullosa simplex with muscular dystrophy	c.6838G>A	p.Glu2280Lys	3.56E-05	18,29	99	0.007	0.997	1
6023	8	144994694	C	T	C/T	PLEC	Epidermolysis bullosa simplex with muscular	c.9706G>A	p.Glu3236Lys	7.41E-05	22,25	99	0.011	0.996	1

							dystrophy								
6023	9	75403280	G	A	G/A	TMC1	Deafness, autosomal recessive 7	c.910G>A	p.Gly304Arg	1.62E-05	32,29	99	0.063	0.852	1
6025	9	677007	C	T	C/T	KANK1	Cerebral palsy, spastic quadriplegic, 2	c.35C>T	p.Ser12Leu	4.07E-05	27,24	99	0.1	0.058	0.991
6025	X	100652903	C	G	C/G	GLA	Fabry disease	c.1184G>C	p.Gly395Ala	1.12E-05	34,32	99	0.029	0.977	1
6025	16	29824554	A	C	A/C	PRRT2	Seizures, benign familial infantile, 2	c.179A>C	p.Asp60Ala	1.63E-05	25,33	99	0	0.393	0.866
6025	9	712110	G	T	G/T	KANK1	Cerebral palsy, spastic quadriplegic, 2	c.1344G>T	p.Met448Ile	0	38,33	99	0.435	0	1
6025	3	193364934	G	A	G/A	OPA1	Optic atrophy plus syndrome	c.1835G>A	p.Arg612Gln	1.22E-05	32,27	99	0.05	0.118	1
6025	8	144999098	CCTT	C	CCTT/C	PLEC	Epidermolysis bullosa simplex, ogna type	c.5407_5409del AAG	p.Lys1803del	1.54E-05	27,20	99	n/a	n/a	n/a
6025	12	7045879	CCAGC AA	C	CCAGCAA /C	ATN1	Dentatorubral-pallidoluysian atrophy	c.1461_1466del ACAGCA	p.Gln488_Gln 489del	8.58E-06	22,13	99	n/a	n/a	n/a
6025	9	126132785	G	C	G/C	CRB2	Ventriculomegaly with cystic kidney disease	c.1453G>C	p.Glu485Gln	3.37489E-04	23,24	99	0.451	0.745	1
6025	9	126129579	G	A	G/A	CRB2	Ventriculomegaly with cystic kidney disease	c.883G>A	p.Ala295Thr	3.32746E-04	27,24	99	0.2	0.001	1
6025	20	22562802	G	C	G/C	FOXA2	Hyperinsulinism, Hypopituitarism with Craniofacial and Endoderm-Derived Organ Abnormalities	c.1078C>G	p.His360Asp	0	18,25	99	0	0.107	1
6025	19	40901953	G	A	G/A	PRX	Hypertrophic neuropathy of dejerine-sottas	c.2306C>T	p.Pro769Leu	8.54E-05	26,24	99	0.006	0.019	1
6025	3	48625289	C	T	C/T	COL7A1	Epidermolysis bullosa dystrophica, autosomal dominant	c.2794G>A	p.Val932Met	2.04E-05	27,31	99	0.049	0.115	1
6025	15	66995828	C	T	C/T	SMAD6	Aortic valve disease 2	c.232C>T	p.Gln78*	0.00E+01	26,26	99	n/a	n/a	1
6027	1	160260377	G	A	G/A	COPA	Autoimmune interstitial lung, joint, and kidney disease	c.3547C>T	p.Arg1183Trp	4.87E-05	35,30	99	0.001	0.551	0.995
6027	8	72182037	C	T	C/T	EYA1	Branchiootorenal syndrome 1	c.988G>A	p.Asp330Asn	0	27,28	99	0	0.998	1
6027	6	7572245	C	G	C/G	DSP	Cardiomyopathy, dilated, with woolly hair, keratoderma, and tooth agenesis	c.2074C>G	p.Pro692Ala	4.07E-05	25,32	99	0.376	0	1
6027	5	89990237	A	G	A/G	ADGRV1	Febrile seizures, familial, 4	c.7664A>G	p.Lys2555Arg	1.22E-05	30,31	99	0.188	0.015	0.628
6027	22	36714278	C	T	C/T	MYH9	Epstein syndrome	c.1201G>A	p.Val401Ile	4.88E-05	13,27	99	0.002	0.961	1
6027	21	47532049	C	T	C/T	COL6A2	Ullrich congenital muscular dystrophy 1	c.272C>T	p.Ala91Val	2.03E-05	27,21	99	0.534	0.005	0.998
6027	1	209796881	G	A	G/A	LAMB3	Epidermolysis bullosa, junctional, herlitz type	c.2327C>T	p.Ser776Leu	7.73E-05	25,28	99	0.011	0.267	0.935
6027	1	209790915	C	T	C/T	LAMB3	Epidermolysis bullosa, junctional, herlitz type	c.3068G>A	p.Arg1023Gln	2.15325E-04	17,24	99	0.329	0.045	1

6027	19	48946427	A	G	A/G	GRIN2D	Epileptic encephalopathy, early infantile, 46	c.3244A>G	p.Thr1082Ala	0	10,19	99	1	0	1
6027	10	112541506	G	A	G/A	RBM20	Cardiomyopathy, dilated, 1dd	c.1139G>A	p.Arg380Gln	1.34E-05	28,23	99	0.081	0.44	1
6027	10	102747996	C	A	C/A	TWNK	Progressive external ophthalmoplegia with mitochondrial dna deletions, autosomal dominant 3	c.29C>A	p.Pro10His	8.13E-06	26,15	99	0.041	0.421	0.981
6027	17	44046571	G	T	G/T	MAPT	Supranuclear palsy, progressive, 1	c.174G>T	p.Leu58Phe	0	32,34	99	n/a	n/a	n/a
6032	3	119121244	G	A	G/A	ARHGAP31	Adams-Oliver syndrome 1	c.1645G>A	p.Ala549Thr	8.35E-06	23,23	99	0.004	0.544	0.837
6032	2	47656891	G	T	G/T	MSH2	Lynch syndrome i	c.1087G>T	p.Val363Leu	2.03E-05	17,25	99	0	0.737	1
6032	16	56362636	G	A	G/A	GNAO1	Neurodevelopmental disorder with involuntary movements	c.397G>A	p.Gly133Ser	8.12E-06	27,18	99	0.607	0.001	0.998
6032	1	8420695	A	G	A/G	RERE	Neurodevelopmental disorder with or without anomalies of the brain, eye, or heart	c.2872T>C	p.Ser958Pro	0	16,12	99	0.003	0.011	0.976
6032	19	14884764	A	G	A/G	ADGRE2	Vibratory urticaria	c.185T>C	p.Met62Thr	3.25E-05	17,8	99	0.387	0	1
6032	11	47354422	C	T	C/T	MYBPC3	Cardiomyopathy, familial hypertrophic, 4	c.3433G>A	p.Val1145Ile	0	23,25	99	0.108	0.696	0.689
6032	2	128177508	G	A	G/A	PROC	Thrombophilia due to protein c deficiency, autosomal dominant	c.53G>A	p.Ser18Asn	0	30,23	99	n/a	n/a	n/a
6032	5	138629712	G	GCGGAGGTGAGCGGTC	G/GCGGAGGTGAGCGGTC	MATR3	Amyotrophic lateral sclerosis 21	c.47_61dupAGGTGAGCGGTCCGG	p.Glu16_Pro20dup	0	19,24	99	n/a	n/a	n/a
6032	4	55156540	C	T	C/T	PDGFRA	Gastrointestinal stromal tumor	c.2221C>T	p.Arg741Cys	8.13E-06	27,22	99	0.073	0.008	0.89
6033	10	50747005	C	A	C/A	ERCC6	Cockayne syndrome b	c.-15+3G>T		0	20,22	99	n/a	n/a	n/a
6033	10	50708686	C	T	C/T	ERCC6	Cockayne syndrome b	c.1583G>A	p.Gly528Glu	0	24,20	99	0	0.997	1
6033	15	90320492	G	C	G/C	MESP2	Spondylocostal dysostosis 2, autosomal recessive	c.904G>C	p.Glu302Gln	0	33,20	99	0	0.96	1
6033	15	90319989	A	T	A/T	MESP2	Spondylocostal dysostosis 2, autosomal recessive	c.401A>T	p.His134Leu	0	16,26	99	0.006	0.951	0.997
6033	11	101353863	A	T	A/T	TRPC6	Focal segmental glomerulosclerosis 2	c.1327T>A	p.Phe443Ile	0	21,27	99	0	0.999	1
6033	8	94768004	A	T	A/T	TMEM67	COACH syndrome	c.224-2A>T		9.43758E-04	10,3	84	n/a	n/a	1
6033	1	74901763	G	T	G/T	TNNI3K	Cardiac conduction disease with or without dilated cardiomyopathy	c.2115-1G>T		0	12,4	72	n/a	n/a	1
6033	14	50050395	T	A	T/A	RPS29	Diamond-Blackfan anemia 13	c.163-2A>T		0	28,11	99	n/a	n/a	1
6033	2	179447931	C	T	C/T	TTN	Lethal congenital contracture syndrome, TTN-related	c.65599G>A	p.Val21867Met	3.33E-05	23,20	99	0.045	0.394	0.984

6033	2	179413493	T	A	T/A	TTN	Lethal congenital contracture syndrome, TTN-related	c.92860A>T	p.Thr30954Ser	2.04E-05	22,27	99	0.11	0.055	0.983
6033	2	179413492	G	A	G/A	TTN	Lethal congenital contracture syndrome, TTN-related	c.92861C>T	p.Thr30954Ile	3.67E-05	22,27	99	0.467	0.018	0.83
6033	1	1469358	C	T	C/T	ATAD3A	Harel-Yoon syndrome	c.1811C>T	p.Thr604Ile	3.25E-05	15,16	99	0.18	0.017	1
6033	1	1464604	C	T	C/T	ATAD3A	Harel-Yoon syndrome	c.1651C>T	p.Arg551Cys	2.85634E-04	17,18	99	0.004	0.726	1
6033	17	29324535	G	A	G/A	RNF135	Macrocephaly, macrosomia, and facial dysmorphism syndrome	c.244G>A	p.Ala82Thr	0	27,24	99	n/a	n/a	n/a
6033	16	89598397	T	C	T/C	SPG7	Spastic paraplegia 7	c.1073T>C	p.Leu358Pro	0	17,22	99	0	1	1
6033	12	65564025	G	C	G/C	LEMD3	Buschke-ollendorff syndrome	c.649G>C	p.Val217Leu	6.76E-06	22,14	99	0.406	0.002	1
6033	11	58919949	A	G	A/G	FAM111A	Gracile bone dysplasia	c.808A>G	p.Lys270Glu	0	27,19	99	0.113	0.086	1
6033	10	61832316	C	T	C/T	ANK3	Mental retardation, autosomal dominant, ANK3-related	c.8323G>A	p.Asp2775Asn	1.63E-05	22,18	99	0.001	0	1
6033	7	152055717	T	A	T/A	KMT2C	Kleefstra syndrome 2	c.205A>T	p.Met69Leu	4.07E-05	19,23	99	0.004	0.011	0.511
6033	5	14485325	A	G	A/G	TRIO	Mental retardation, autosomal dominant 44	c.6805A>G	p.Ile2269Val	4.22E-06	24,16	99	0.985	0.29	1
6033	4	6302753	T	A	T/A	WFS1	Wolfram-like syndrome, autosomal dominant	c.1231T>A	p.Ser411Thr	0	15,22	99	0.012	0.942	1
6033	2	21236313	A	G	A/G	APOB	Hypobetalipoproteinemia, familial, 1	c.3935T>C	p.Met1312Thr	2.03E-05	21,32	99	0.006	0.053	0.969
6033	1	231557316	C	A	C/A	EGLN1	Erythrocytosis, familial, 3	c.319G>T	p.Ala107Ser	3.30E-05	10,14	99	0.772	0.01	1
6033	8	94768003	T	TTTTTTTTTTTTTTTTTTTTTTCC	T/TTTTTTTTTTTTTTTTTTTTTTCC	TMEM67	COACH syndrome	c.224-3_224-2insTTTTTTTTTTTTTTTTTTTTTTCC		0	10,3	84	n/a	n/a	n/a
6033	18	31325896	CTTGGCTTTG	C	CTTGGCTTTG/C	ASXL3	Bainbridge-Ropers syndrome	c.6085_6093delTTGGCTTTG	p.Leu2029_Leu2031del	4.32E-05	26,7	99	n/a	n/a	n/a
6033	16	30991353	G	A	G/A	SETD1A	Schizophrenia, SETD1A-related	c.4246G>A	p.Glu1416Lys	9.11E-06	19,12	99	0.596	0.003	0.993
6033	3	132169545	G	A	G/A	DNAJC13	Parkinson disease 21	c.391G>A	p.Glu131Lys	0	22,23	99	0.254	0.13	1
6033	3	37090455	T	C	T/C	MLH1	Muir-torre syndrome	c.2050T>C	p.Tyr684His	0	32,19	99	0	0.996	1
6033	2	47709932	T	G	T/G	MSH2	Muir-torre syndrome	c.2649T>G	p.Ile883Met	8.13E-06	27,23	99	0.127	0.591	0.999
6034	10	97387319	G	C	G/C	ALDH18A1	Cutis laxa, autosomal dominant 3	c.958C>G	p.Gln320Glu	0	31,33	99	0.823	0.006	1
6034	19	39034013	A	T	A/T	RYR1	Central core disease of muscle	c.11716A>T	p.Thr3906Ser	0	32,17	99	0.033	0.445	0.995
6034	22	36745262	T	C	T/C	MYH9	Epstein syndrome	c.20A>G	p.Asp7Gly	0	14,15	99	0.01	0.071	1
6034	4	77662410	G	C	G/C	SHROOM3	Neural tube defect, SHROOM3-related	c.3084G>C	p.Glu1028Asp	1.29E-05	19,23	99	0.002	0.477	0.781
6034	15	74637532	C	T	C/T	CYP11A1	arrhythmogenic right ventricular dysplasia,	c.478G>A	p.Ala160Thr	3.66E-05	33,17	99	0.302	0.066	1

							familial, 13								
6034	6	31936719	G	A	G/A	SKIV2L	Trichohepatoenteric syndrome 2	c.3252G>A	p.Met1084Ile	2.24131E-04	26,28	99	0.373	0.026	0.981
6034	6	31931858	C	T	C/T	SKIV2L	Trichohepatoenteric syndrome 2	c.1816C>T	p.Arg606Cys	2.20325E-04	24,20	99	0.002	0.663	1
6034	14	50623817	G	A	G/A	SOS2	Noonan syndrome 9	c.1957C>T	p.Pro653Ser	0	20,22	99	0.378	0.137	1
6034	12	14798171	T	G	T/G	GUCY2C	Diarrhea 6	c.1789A>C	p.Ile597Leu	8.27E-06	26,36	99	0.005	0.162	1
6034	11	47369415	G	A	G/A	MYBPC3	Cardiomyopathy, familial hypertrophic, 4	c.814C>T	p.Arg272Cys	4.66E-05	28,22	99	0	0.836	1
6034	10	68979459	T	C	T/C	CTNNA3	Arrhythmogenic right ventricular dysplasia, familial, 13	c.749A>G	p.Asn250Ser	1.62E-05	33,32	99	0.149	0.042	0.971
6034	6	152774695	G	A	G/A	SYNE1	Emery-dreifuss muscular dystrophy 4, autosomal dominant	c.1754C>T	p.Pro585Leu	1.62E-05	17,30	99	0	0	1
6035	22	50962500	C	T	C/T	SCO2	Myopia 6	c.341G>A	p.Arg114His	8.14578E-04	21,22	99	0.019	0.958	1
6035	16	16313412	G	A	G/A	ABCC6	Pseudoxanthoma elasticum	c.473C>T	p.Ala158Val	0.0011749	9,6	99	0.231	0.124	0.999
6035	16	16248912	C	T	C/T	ABCC6	Pseudoxanthoma elasticum	c.3883-24G>A		0.00616609	16,15	99	n/a	n/a	n/a
6035	16	16243952	C	T	C/T	ABCC6	Pseudoxanthoma elasticum	c.*38G>A		0.00826398	12,16	99	n/a	n/a	n/a
6035	2	166245829	A	C	A/C	SCN2A	Epileptic encephalopathy, early infantile, 11	c.5513A>C	p.Gln1838Pro	0	17,24	99	0.002	0.936	1
6035	1	120458477	C	T	C/T	NOTCH2	Hajdu-Cheney syndrome	c.6868G>A	p.Glu2290Lys	3.25E-05	13,20	99	0.804	0.002	0.693
6035	9	117826952	G	C	G/C	TNC	Deafness, autosomal dominant 56	c.3461C>G	p.Pro1154Arg	1.22E-05	17,21	99	0.289	0.012	1
6035	7	19156668	T	TGCCGCCGCCGCCGCCCGCGCC	T/TGCCGCCGCCGCCGCCCGCGCC	TWIST1	Saethre-Chotzen syndrome	c.256_276dupGGCGCGGGCGGCGGCGGCGGC	p.Gly92_Ser93insGlyAlaGlyGlyGlyGlyGly	1.60E-05	13,14	99	n/a	n/a	n/a
6035	19	10305557	G	A	G/A	DNMT1	Cerebellar ataxia, deafness, and narcolepsy, autosomal dominant	c.19C>T	p.Pro7Ser	1.73E-05	18,17	99	0.013	0.998	0.829
6036	5	125930860	G	C	G/C	ALDH7A1	Epilepsy, pyridoxine-dependent	c.31C>G	p.His11Asp	1.24665E-04	17,19	99	0.022	0.002	1
6036	5	125930858	G	C	G/C	ALDH7A1	Epilepsy, pyridoxine-dependent	c.33C>G	p.His11Gln	1.30531E-04	17,19	99	0.266	0.001	1
6036	16	29810653	T	TGGGTCAGAGGACAACC	T/TGGGTCAGAGGACAACC	KIF22	Spondyloepimetaphyseal dysplasia with joint laxity, type 2	c.831_846dupGTCAGAGGACAACCGG	p.Arg283fs	0	18,18	99	n/a	n/a	n/a
6036	2	238249419	C	A	C/A	COL6A3	Ullrich congenital muscular dystrophy 1	c.8140G>T	p.Ala2714Ser	3.25E-05	26,17	99	0.131	0.02	0.724
6036	20	57415862	C	T	C/T	GNAS	Pseudohypoparathyroidism, type Ia	c.701C>T	p.Pro234Leu	4.14E-06	19,21	99	0.005	0.999	0.946
6036	2	121745793	T	C	T/C	GLI2	Holoprosencephaly 9	c.2303T>C	p.Leu768Pro	4.48E-06	10,20	99	0.02	0.936	1
6036	5	41160262	A	G	A/G	C6	Complement component 6 deficiency	c.1666T>C	p.Ser556Pro	1.63E-05	25,27	99	0.022	0.282	0.997

6036	20	60908237	A	G	A/G	LAMA5	Extracellular matrix syndrome, LAMA5-related	c.3191T>C	p.Leu1064Pro	0	22,21	99	0.37	0.454	1
6036	18	24127680	C	T	C/T	KCTD1	Scalp-ear-nipple syndrome	c.821G>A	p.Gly274Asp	0	15,24	99	n/a	n/a	n/a
6036	16	56996930	G	A	G/A	CETP	Hyperalphalipoproteinemia 1	c.127G>A	p.Glu43Lys	2.03E-05	27,29	99	0.25	0.564	1
6036	11	47367827	C	T	C/T	MYBPC3	Cardiomyopathy, familial hypertrophic, 4	c.1021G>A	p.Gly341Ser	4.88E-05	26,29	99	0.001	0.953	1
6036	5	60822215	A	C	A/C	ZSWIM6	Neurodevelopmental disorder with movement abnormalities, abnormal gait, and autistic features	c.1829A>C	p.Gln610Pro	2.79E-05	24,26	99	0.005	0.622	1
6036	2	21235429	G	C	G/C	APOB	Hypercholesterolemia, due to ligand-defective apo B	c.4311C>G	p.Phe1437Leu	4.47E-05	26,17	99	0.55	0.002	0.593
6036	1	169483637	T	C	T/C	F5	Thrombophilia due to activated protein c resistance	c.6604A>G	p.Ile2202Val	7.73E-05	24,26	99	0.117	0.169	0.828
6036	15	100252735	AGCAGCAGCC	A	AGCAGCAGCC/A	MEF2A	Coronary artery disease, autosomal dominant, 1	c.1283_1291delAGCAGCCGC	p.Gln428_Pro430del	6.83E-06	17,13	99	n/a	n/a	n/a
6036	7	128484814	G	A	G/A	FLNC	Myopathy, myofibrillar, 5	c.3295G>A	p.Val1099Ile	3.25E-05	22,26	99	0.289	0.527	1
6037	12	110222199	TG	T	TG/T	TRPV4	Metatropic dysplasia	c.2379delC	p.Ile794fs	0	26,22	99	n/a	n/a	n/a
6037	18	42532087	C	G	C/G	SETBP1	Schinzel-giedion midface retraction syndrome	c.2782C>G	p.Leu928Val	4.07E-06	22,20	99	0.006	0.941	0.994
6037	6	33134580	T	G	T/G	COL11A2	Otospondylomegaepiphyseal dysplasia, autosomal dominant	c.4255A>C	p.Ile1419Leu	8.29E-06	17,22	99	0.012	0.033	0.988
6037	16	89346613	C	T	C/T	ANKRD11	Kbg syndrome	c.6337G>A	p.Gly2113Ser	3.10E-05	19,18	99	0.426	0.005	1
6037	4	77357261	C	T	C/T	SHROOM3	Neural tube defect, SHROOM3-related	c.56C>T	p.Thr19Met	8.13E-06	24,29	99	0.11	0.003	1
6037	2	179649037	C	A	C/A	TTN	Hereditary myopathy with early respiratory failure	c.2535G>T	p.Glu845Asp	4.89E-05	31,24	99	0	0.978	0.98
6037	2	149227824	G	A	G/A	MBD5	Mental retardation, autosomal dominant 1	c.2312G>A	p.Ser771Asn	4.07E-06	18,19	99	0.113	0	0.994
6037	1	223177531	A	C	A/C	DISP1	Holoprosencephaly, DISP1-related	c.2792A>C	p.Tyr931Ser	2.84E-05	28,20	99	0.065	0.056	0.997
6037	X	49807059	C	G	G	CLCN5	Dent disease 1	c.151C>G	p.Arg51Gly	0	0,26	78	0.753	0.149	0.893
6037	12	6091119	A	G	A/G	VWF	Von willebrand disease, type 2	c.7120T>C	p.Ser2374Pro	2.47466E-04	25,7	99	0.104	0.216	1
6037	12	6091106	T	G	T/G	VWF	Von willebrand disease, type 2	c.7133A>C	p.His2378Pro	0	22,9	99	0.054	0.942	1
6038	11	46761055	G	A	G/A	F2	Thrombophilia due to thrombin defect	c.*97G>A		0.00839631	22,25	99	n/a	n/a	n/a
6038	11	119077312	T	A	T/A	CBL	Noonan syndrome-like disorder with or without juvenile myelomonocytic leukemia	c.185T>A	p.Leu62His	7.00E-06	20,20	99	0.008	0.998	1
6038	20	44054440	C	T	C/T	PIGT	Paroxysmal nocturnal hemoglobinuria 2	c.1711C>T	p.Arg571Cys	1.63E-05	21,18	99	0.001	0.963	1

6038	14	91747827	A	G	A/G	CCDC88C	Hydrocephalus, nonsyndromic, autosomal recessive 1	c.4673T>C	p.Phe1558Ser	3.77668E-04	16,20	99	0.518	0.002	1
6038	14	91739760	C	T	C/T	CCDC88C	Hydrocephalus, nonsyndromic, autosomal recessive 1	c.5296G>A	p.Val1766Met	7.69455E-04	14,23	99	0.237	0.004	1
6038	19	39217717	C	T	C/T	ACTN4	Focal segmental glomerulosclerosis 1	c.2311C>T	p.Arg771Trp	1.22E-05	24,25	99	0	1	1
6038	2	21234578	A	G	A/G	APOB	Hypobetalipoproteinemia, familial, 1	c.5162T>C	p.Val1721Ala	0	23,25	99	0.861	0.004	1
6038	3	183887940	C	T	C/T	DVL3	Robinow syndrome, autosomal dominant 3	c.1645C>T	p.Pro549Ser	3.29E-05	25,33	99	0.791	0.992	1
6038	3	53835340	G	C	G/C	CACNA1D	Sinoatrial node dysfunction and deafness	c.5356G>C	p.Ala1786Pro	2.19286E-04	27,21	99	0.101	0.036	0.843
6038	3	53757972	A	C	A/C	CACNA1D	Sinoatrial node dysfunction and deafness	c.2106A>C	p.Gln702His	0	18,19	99	0.112	0.029	0.833
6038	6	111701342	T	A	T/A	REV3L	Moebius syndrome, REV3L-related	c.1297A>T	p.Met433Leu	0	20,18	99	0.073	0.002	1
6040	16	3808051	T	A	T/A	CREBBP	Intellectual disability, syndromic, CREBBP-related	c.3370-2A>T		0	19,8	99	n/a	n/a	1
6040	16	3808050	C	A	C/A	CREBBP	Intellectual disability, syndromic, CREBBP-related	c.3370-1G>T		0	19,8	99	n/a	n/a	1
6040	10	95549851	TCCAG GAG	T	TCCAGGA G/T	LGI1	Epilepsy, familial temporal lobe, 1	c.432-2_436delAGGA GCC	p.Leu144fs	2.13E-05	10,5	99	n/a	n/a	n/a
6040	10	95549860	C	T	C/T	LGI1	Epilepsy, familial temporal lobe, 1	c.436C>T	p.Leu146Phe	0	11,5	99	0	0.995	1
6040	1	202699104	GT	G	GT/G	KDM5B	Neurodevelopmental disorder, KDM5B-related	c.4335delA	p.Lys1445fs	0	31,22	99	n/a	n/a	n/a
6040	5	90012539	G	A	G/A	ADGRV1	Usher syndrome, type 2C	c.9440G>A	p.Arg3147Gln	0.00028922	32,26	99	0.095	0.007	1
6040	5	89925235	G	T	G/T	ADGRV1	Usher syndrome, type 2C	c.1718G>T	p.Gly573Val	0.00028453	23,14	99	0.004	0.976	1
6040	2	179647623	C	T	C/T	TTN	Lethal congenital contracture syndrome, TTN-related	c.3010G>A	p.Glu1004Lys	1.26205E-04	21,17	99	0.378	0.996	0.999
6040	2	179544340	G	A	G/A	TTN	Lethal congenital contracture syndrome, TTN-related	c.33650C>T	p.Ala11217Va l	7.80E-05	21,33	99	0.7	0	1
6040	2	179446251	A	T	A/T	TTN	Lethal congenital contracture syndrome, TTN-related	c.66744T>A	p.Asp22248Gl u	0	23,25	99	0.686	0.001	0.788
6040	2	179421725	T	A	T/A	TTN	Lethal congenital contracture syndrome, TTN-related	c.88156A>T	p.Ser29386Cy s	7.33E-05	29,21	99	0.004	0.947	1
6040	2	179411173	C	T	C/T	TTN	Lethal congenital contracture syndrome, TTN-related	c.94885G>A	p.Ala31629Th r	2.04E-05	19,25	99	0.014	0.999	1
6040	21	47402652	C	T	C/T	COL6A1	Ullrich congenital muscular dystrophy 1	c.202C>T	p.Arg68Cys	5.49585E-04	15,18	99	0.01	0.987	1
6040	9	439282	C	T	C/T	DOCK8	Mental retardation,	c.5117C>T	p.Ser1706Phe	3.25E-05	21,17	99	0	0.993	1

							autosomal dominant 2								
6040	10	95549863	G	T	G/T	LGI1	Epilepsy, familial temporal lobe, 1	c.439G>T	p.Ala147Ser	4.40E-06	12,5	99	0.066	0.962	1
6040	12	124242475	G	GTTTT	G/GTTTT	ATP6V0A2	Cutis laxa, autosomal recessive, type iia	c.2468_2469ins TTTT	p.Glu824fs	9.04E-06	18,5	99	n/a	n/a	n/a
6040	12	124242471	TAGG G	T	TAGGG/T	ATP6V0A2	Cutis laxa, autosomal recessive, type iia	c.2466-2_2467delAGG G	p.Trp822fs	8.84E-06	18,5	99	n/a	n/a	n/a
6040	12	125437068	T	G	T/G	DHX37	Neurodevelopmental disorder with microcephaly, seizures, and cortical atrophy	c.2744A>C	p.Gln915Pro	0	19,5	87	0.465	0.001	0.871
6040	12	125437066	G	C	G/C	DHX37	Neurodevelopmental disorder with microcephaly, seizures, and cortical atrophy	c.2746C>G	p.Pro916Ala	0	19,5	87	0.025	1	1
6040	19	48725063	G	A	G/A	CARD8	Crohn’s disease like inflammation, CARD8-related	c.1085C>T	p.Ser362Leu	2.85E-05	27,17	99	0.004	0.988	1
6043	17	8457273	A	G	A/G	MYH10	Multiple congenital anomalies, MYH10-related	c.820T>C	p.Tyr274His	0	20,21	99	0	0.369	1
6043	7	39472788	A	T	A/T	POU6F2	Wilms tumor 5	c.1139A>T	p.Gln380Leu	3.23E-05	11,16	99	0	0.953	1
6043	9	131378117	C	G	C/G	SPTAN1	Epileptic encephalopathy, early infantile, 5	c.5355C>G	p.Ile1785Met	0	16,18	99	0	1	1
6043	12	5020587	G	T	G/T	KCNA1	Episodic ataxia, type 1	c.43G>T	p.Ala15Ser	2.89E-05	21,22	99	0.615	0.005	1
6043	9	35065348	C	T	C/T	VCP	Inclusion body myopathy with early-onset paget disease with or without frontotemporal dementia 1	c.476G>A	p.Arg159His	8.12E-06	17,17	99	0.095	0.144	1
6043	13	50070187	G	T	G/T	PHF11	Ige responsiveness, atopic	c.50G>T	p.Ser17Ile	3.25E-05	19,19	99	0.072	0.047	1
6043	7	73470711	C	T	C/T	ELN	Cutis laxa, autosomal dominant 1	c.1261C>T	p.Pro421Ser	0	18,15	99	0.054	0.299	1
6043	6	111652932	A	G	A/G	REV3L	Moebius syndrome, REV3L-related	c.7981T>C	p.Tyr2661His	1.22E-05	25,15	99	0.078	0.246	0.999
6043	6	16327700	A	G	A/G	ATXN1	Spinocerebellar ataxia 1	c.842T>C	p.Leu281Pro	0	18,11	99	0	0.577	1
6043	1	152276444	A	C	A/C	FLG	Ichthyosis vulgaris	c.10918T>G	p.Ser3640Ala	2.47E-05	16,4	99	0.182	0.948	1
6046	8	27320780	G	A	G/A	CHRNA2	Epilepsy, nocturnal frontal lobe, 4	c.1180C>T	p.Arg394Cys	1.26E-05	18,15	99	0.026	0.231	1
6046	8	41836199	A	T	A/T	KAT6A	Mental retardation, autosomal dominant 32	c.1004T>A	p.Ile335Lys	0	27,34	99	0	0.623	1
6046	4	114195696	C	T	C/T	ANK2	Cardiac arrhythmia, ankyrin-b-related	c.1574C>T	p.Ala525Val	4.07E-06	23,30	99	0.156	0.997	1
6046	19	38981295	A	G	A/G	RYR1	Central core disease of muscle	c.6050A>G	p.Asp2017Gly	4.06E-06	27,29	99	0.001	0.003	0.957
6046	9	140706000	G	A	G/A	EHMT1	Kleefstra syndrome	c.2800G>A	p.Ala934Thr	8.25E-06	25,28	99	0.086	0.771	1
6046	11	77824929	T	A	T/A	ALG8	Congenital disorder of glycosylation, type Ih	c.777+3A>T		0	20,7	99	n/a	n/a	n/a

6046	11	77824928	T	A	T/A	ALG8	Congenital disorder of glycosylation, type Ih	c.777+4A>T		0	20,7	99	n/a	n/a	n/a
6046	10	88854304	C	G	C/G	GLUD1	Hyperinsulinemic hypoglycemia, familial, 6	c.223G>C	p.Asp75His	0	23,17	99	0.628	0.017	1
6047	12	112920008	A	G	A/G	PTPN11	Noonan syndrome 1	c.1223A>G	p.Gln408Arg	0	23,22	99	0.403	0.017	1
6047	2	228124590	C	T	C/T	COL4A3	Hematuria, benign familial	c.1111C>T	p.Gln371*	0	31,25	99	n/a	n/a	1
6047	5	136961504	T	C	T/C	KLHL3	Pseudohypoaldosteronism, type iid	c.1673A>G	p.Asn558Ser	4.87E-05	20,26	99	0.157	0.021	1
6047	14	24709516	A	ATGTAG CAAT	A/ATGTA GCAAT	TINF2	Dyskeratosis congenita, autosomal dominant 3	c.1073_1081du pATTGCTACA	p.Tyr360_Met 361insAsnCys Tyr	4.06E-05	22,24	99	n/a	n/a	n/a
6047	17	63554000	C	G	C/G	AXIN2	Colorectal cancer	c.739G>C	p.Val247Leu	2.03E-05	36,20	99	0.34	0.54	1
6047	8	95390571	A	T	A/T	RAD54B	Colorectal cancer	c.2352T>A	p.Ser784Arg	1.22E-05	25,30	99	0.016	0.926	1
6047	16	89261338	G	C	G/C	CDH15	Mental retardation, autosomal dominant 3	c.2220G>C	p.Glu740Asp	1.27E-05	21,21	99	0	1	0.998
6047	19	38985027	C	T	C/T	RYR1	Central core disease of muscle	c.6310C>T	p.Arg2104Cys	1.24E-05	20,15	99	0.106	0.804	0.999
6047	8	145006317	G	A	G/A	PLEC	Epidermolysis bullosa simplex with muscular dystrophy	c.2474C>T	p.Pro825Leu	1.94405E-04	24,21	99	0.002	0.996	1
6047	8	144994166	G	A	G/A	PLEC	Epidermolysis bullosa simplex with muscular dystrophy	c.10234C>T	p.Arg3412Cys	2.44E-05	27,12	99	0	0.809	1
6047	8	144992495	C	T	C/T	PLEC	Epidermolysis bullosa simplex with muscular dystrophy	c.11905G>A	p.Asp3969Asn	0	20,30	99	0.058	0.366	1
6047	21	47549355	G	T	G/T	COL6A2	Ullrich congenital muscular dystrophy 1	c.2707G>T	p.Val903Leu	4.52E-05	13,23	99	0.045	0.66	1
6047	19	54652195	A	G	A/G	CNOT3	Neurodevelopmental disorder, CNOT3-related	c.1207A>G	p.Ser403Gly	2.65E-05	14,17	99	0.582	0	1
6047	17	78261842	T	C	T/C	RNF213	Moyamoya disease 2	c.637T>C	p.Ser213Pro	4.67315E-04	13,22	99	0.326	0.002	1
6047	12	32871592	C	G	C/G	DNM1L	Encephalopathy due to defective mitochondrial and peroxisomal fission 1	c.674C>G	p.Ala225Gly	4.06E-05	18,22	99	1	0.248	1
6047	7	150642539	G	C	G/C	KCNH2	Long qt syndrome 2	c.3394C>G	p.Pro1132Ala	1.88E-05	29,15	99	0.022	0.111	0.938
6047	7	103202098	G	C	G/C	RELN	Epilepsy, familial temporal lobe, 7	c.5410C>G	p.Leu1804Val	4.07E-06	20,23	99	0.008	0.832	1
6047	X	136649006	CGCCG CT	C	C	ZIC3	Heterotaxy, visceral, 1, x-linked	c.160_165delGC TGCC	p.Ala54_Ala5 5del	4.63E-05	1,28	46	n/a	n/a	n/a
6047	17	12903577	G	A	G/A	ELAC2	Prostate cancer, hereditary, 2	c.1319C>T	p.Thr440Ile	2.44E-05	34,20	99	0.182	0.005	1
6049	11	46761055	G	A	G/A	F2	Thrombophilia due to thrombin defect	c.*97G>A		0.00839631	19,14	99	n/a	n/a	n/a
6049	3	50385316	C	T	C/T	NPRL2	Epilepsy, familial focal, with variable foci 2	c.949G>A	p.Gly317Arg	0	24,21	99	0.001	0.999	1
6049	X	63411722	T	A	A	AMER1	Osteopathia striata with	c.1445A>T	p.Asp482Val	0	0,19	57	0.02	0.915	1

							cranial sclerosis								
6049	2	179600385	G	C	G/C	TTN	Lethal congenital contracture syndrome, TTN-related	c.14788C>G	p.Pro4930Ala	1.14063E-04	21,20	99	0.242	0.003	1
6049	2	179429702	A	T	A/T	TTN	Lethal congenital contracture syndrome, TTN-related	c.81157T>A	p.Tyr27053Asn	1.06573E-04	19,21	99	0	0.93	1
6049	2	69472500	CAG	C	CAG/C	ANTXR1	GAPO syndrome	c.1579_1580delAG	p.Ser527fs	2.36E-05	7,5	99	n/a	n/a	n/a
6049	2	69472482	CTG	C	CTG/C	ANTXR1	GAPO syndrome	c.1561_1562delTG	p.Cys521fs	0	10,10	99	n/a	n/a	n/a
6049	2	69472472	CTG	C	CTG/C	ANTXR1	GAPO syndrome	c.1551_1552delTG	p.His520fs	0	10,10	99	n/a	n/a	n/a
6049	2	69472460	CT	C	CT/C	ANTXR1	GAPO syndrome	c.1539delT	p.Pro515fs	0	8,10	99	n/a	n/a	n/a
6049	9	130374707	G	A	G/A	STXBP1	Epileptic encephalopathy, early infantile, 4	c.25G>A	p.Val9Ile	4.44E-06	11,13	99	0.499	0.002	0.911
6049	2	69472504	G	C	G/C	ANTXR1	GAPO syndrome	c.1582G>C	p.Ala528Pro	0.00011661	8,4	99	0.305	0.012	0.584
6049	2	69472481	A	C	A/C	ANTXR1	GAPO syndrome	c.1559A>C	p.His520Pro	0	10,10	99	0.059	0	0.727
6049	18	31325896	CTTGGCTTTG	C	CTTGGCTTTG/C	ASXL3	Bainbridge-Ropers syndrome	c.6085_6093delTTGGCTTTG	p.Leu2029_Leu2031del	4.32E-05	13,7	99	n/a	n/a	n/a
6049	17	78346922	G	A	G/A	RNF213	Moyamoya disease 2	c.13046G>A	p.Arg4349His	2.04E-05	23,17	99	0.991	0	1
6049	10	117221505	G	A	G/A	ATRNL1	Cognitive impairment, autism and dysmorphic features, ATRNL1-related	c.3377G>A	p.Arg1126His	2.05E-05	19,26	99	0.006	0.163	0.547
6049	3	184910744	C	T	C/T	EHHADH	Fanconi renotubular syndrome 3	c.1442G>A	p.Gly481Glu	2.85E-05	22,25	99	0.001	1	1
6049	2	227958975	A	C	A/C	COL4A4	Alport syndrome, autosomal dominant	c.1235T>G	p.Phe412Cys	1.63E-05	30,22	99	0.174	0.482	1
6049	1	32193803	G	A	G/A	ADGRB2	Progressive spastic paraparesis, BAI2-related	c.4495C>T	p.Arg1499Cys	2.84E-05	20,25	99	0	0.432	0.997
6049	1	27878413	G	A	G/A	AHDC1	Xia-Gibbs syndrome	c.214C>T	p.Arg72Trp	2.24E-05	23,19	99	0.01	0.388	0.806
6049	19	36212153	CTG	C	CTG/C	KMT2B	Dystonia 28, childhood-onset	c.1905_1906delTG	p.Ala636fs	7.45E-06	16,4	99	n/a	n/a	n/a
6049	19	36212135	CGG	C	CGG/C	KMT2B	Dystonia 28, childhood-onset	c.1887_1888delGG	p.Ala630fs	7.86E-06	14,5	99	n/a	n/a	n/a
6049	19	36212117	CAG	C	CAG/C	KMT2B	Dystonia 28, childhood-onset	c.1869_1870delAG	p.Ala624fs	4.69E-05	12,5	99	n/a	n/a	n/a
6049	11	61537817	G	GCCCCCCCCCCCCCCC	G/GCCCCCCCCCCCCCCC	MYRF	Cardiac and urogenital anomalies syndrome, MYRF-related	c.566_567insCCCCCCCCCCCCCC	p.Pro189_Pro190insProProProProPro	0	32,8	99	n/a	n/a	n/a
6049	2	69472510	A	C	A/C	ANTXR1	GAPO syndrome	c.1588A>C	p.Thr530Pro	4.64E-05	7,3	99	0.34	0.094	0.816
6049	12	988763	A	G	A/G	WNK1	Pseudohypoaldosteronism, type iic	c.3892A>G	p.Thr1298Ala	2.84E-05	7,4	99	0.411	0.001	1
6049	1	180842979	G	A	G/A	XPR1	Basal ganglia calcification, idiopathic, 6	c.1709G>A	p.Arg570His	8.12E-06	21,15	99	0	1	1

6050	X	153580342	T	C	T/C	FLNA	Melnick-Needles syndrome	c.6817A>G	p.Ile2273Val	5.60E-06	22,20	99	0.148	0.023	1
6050	12	11803078	C	T	C/T	ETV6	Thrombocytopenia 5	c.17C>T	p.Ala6Val	0	23,12	99	0.08	0.001	0.967
6050	20	30414514	A	AG	A/AG	MYLK2	Cardiomyopathy, familial hypertrophic, 1	c.1080dupG	p.Tyr361fs	8.12E-06	28,19	99	n/a	n/a	n/a
6050	16	2160772	C	T	C/T	PKD1	Polycystic kidney disease 1	c.4396G>A	p.Val1466Met	3.69E-05	22,16	99	0.071	0.373	0.988
6050	12	116445244	G	A	G/A	MED13L	Mental retardation and distinctive facial features with or without cardiac defects	c.2210C>T	p.Thr737Met	2.03E-05	22,21	99	0.004	0.001	0.965
6050	17	78190963	C	T	C/T	SGSH	Mucopolysaccharidosis, type iiia	c.152G>A	p.Arg51His	5.85E-05	26,18	99	n/a	0.001	1
6050	17	78187993	G	T	G/T	SGSH	Mucopolysaccharidosis, type iiia	c.641C>A	p.Ala214Asp	0.000278	13,19	99	n/a	n/a	0.995
6050	19	48946667	C	A	C/A	GRIN2D	Epileptic encephalopathy, early infantile, 46	c.3484C>A	p.Arg1162Ser	3.63E-05	23,18	99	0.003	0.353	0.995
6050	19	42776485	G	T	G/T	CIC	Mental retardation, autosomal dominant 45	c.550G>T	p.Gly184Cys	3.24E-05	17,26	99	n/a	n/a	1
6050	19	10251841	A	G	A/G	DNMT1	Cerebellar ataxia, deafness, and narcolepsy, autosomal dominant	c.3334T>C	p.Phe1112Leu	4.06E-06	16,15	99	0.37	0.944	1
6050	11	77823783	G	C	G/C	ALG8	Polycystic liver disease 3 with or without kidney cysts	c.811C>G	p.Pro271Ala	4.07E-06	16,19	99	0	1	1
6055	15	48707728	C	T	C/T	FBN1	Marfan syndrome	c.8051+5G>A		2.46E-05	24,23	99	n/a	n/a	n/a
6055	10	12154929	G	A	G/A	DHTKD1	Charcot-marie-tooth disease, axonal, type 2q	c.2185G>A	p.Gly729Arg	0.00166476	27,23	99	0.006	0.854	1
6055	3	38892121	G	T	G/T	SCN11A	Episodic pain syndrome, familial, 3	c.4178C>A	p.Ala1393Asp	0	20,22	99	0.241	0	1
6055	X	76938473	T	C	T/C	ATRX	Alpha-thalassemia/mental retardation syndrome, x-linked	c.2275A>G	p.Thr759Ala	0	27,21	99	0.308	0.01	1
6055	2	179427779	G	C	G/C	TTN	Salih myopathy	c.83080C>G	p.Arg27694Gly	0	31,22	99	0.002	0.999	1
6055	17	17698676	C	T	C/T	RAI1	Smith-magenis syndrome	c.2414C>T	p.Ser805Leu	2.99E-05	16,9	99	0.009	0.007	1
6055	3	50387396	T	C	T/C	NPRL2	Epilepsy, familial focal, with variable foci 2	c.136A>G	p.Thr46Ala	0	25,22	99	0.032	0.511	1
6055	13	23911063	C	T	C/T	SACS	Spastic ataxia, charlevoix-saguenay type	c.6952G>A	p.Ala2318Thr	2.07536E-04	24,26	99	0.373	0.728	0.991
6055	13	23909388	T	C	T/C	SACS	Spastic ataxia, charlevoix-saguenay type	c.8627A>G	p.Glu2876Gly	0	19,33	99	0.008	0.814	0.999
6055	2	179596269	G	A	G/A	TTN	Salih myopathy	c.17224C>T	p.Leu5742Phe	2.74419E-04	29,17	99	0.35	0.954	1
6055	2	179401935	C	T	C/T	TTN	Salih myopathy	c.99901G>A	p.Glu33301Lys	2.90841E-04	11,19	99	0.091	0.683	1
6055	16	9858492	C	T	C/T	GRIN2A	Epilepsy, focal, with speech disorder and with or without mental retardation	c.2909G>A	p.Arg970Gln	4.88E-05	29,25	99	0.315	0.739	0.995

6055	7	151860720	C	A	C/A	KMT2C	Kleefstra syndrome 2	c.9942G>T	p.Gln3314His	2.05E-05	18,21	99	0.005	0.556	0.876
6055	5	149449431	C	G	C/G	CSF1R	Leukoencephalopathy, hereditary diffuse, with spheroids	c.1510+5G>C		8.36E-06	14,14	99	n/a	n/a	n/a
6055	1	196799639	G	A	G/A	CFHR1	Hemolytic uremic syndrome, atypical, susceptibility to, 1	c.617G>A	p.Gly206Glu	0	23,17	99	0.003	0.046	1
6055	1	2237598	G	A	G/A	SKI	Shprintzen-goldberg craniosynostosis syndrome	c.1907G>A	p.Arg636His	0	17,21	99	0.044	0.959	1
6057	19	5896427	C	CGGGGG	C/CGGGGG	NDUFA11	Mitochondrial complex I deficiency, autosomal recessive	c.349_350insCCCCC	p.Ser117fs	0	18,9	99	n/a	n/a	n/a
6057	4	151837660	G	C	G/C	LRBA	Immunodeficiency, common variable, 8, with autoimmunity	c.787C>G	p.Leu263Val	7.99426E-04	23,37	99	0.834	0.994	1
6057	4	151509208	C	T	C/T	LRBA	Immunodeficiency, common variable, 8, with autoimmunity	c.6355G>A	p.Asp2119Asn	4.42101E-04	26,36	99	0.026	0.004	1
6057	19	5896445	T	G	T/G	NDUFA11	Mitochondrial complex I deficiency, autosomal recessive	c.332A>C	p.His111Pro	4.72396E-04	15,10	99	n/a	n/a	1
6057	19	5896439	T	G	T/G	NDUFA11	Mitochondrial complex I deficiency, autosomal recessive	c.338A>C	p.His113Pro	4.23E-05	15,11	99	n/a	n/a	1
6057	19	5896428	T	G	T/G	NDUFA11	Mitochondrial complex I deficiency, autosomal recessive	c.349A>C	p.Ser117Arg	0	17,8	99	n/a	n/a	1
6057	16	71149659	C	T	C/T	HYDIN	Ciliary dyskinesia, primary, 5	c.1267G>A	p.Val423Met	4.78E-05	9,8	99	0.003	0.994	0.964
6057	22	21351061	A	G	A/G	LZTR1	Noonan syndrome 10	c.2296A>G	p.Met766Val	1.63E-05	15,23	99	0.306	0.027	1
6057	18	10857098	G	A	G/A	PIEZO2	Marden-walker syndrome	c.604C>T	p.Arg202Cys	3.54E-05	27,25	99	0.011	n/a	1
6057	10	102587341	C	G	C/G	PAX2	Papillorenal syndrome	c.1150C>G	p.Pro384Ala	1.23E-05	14,16	99	0.127	0.981	0.992
6057	3	9976153	C	T	C/T	CRELD1	Atrioventricular septal defect, partial, with heterotaxy syndrome	c.31C>T	p.Pro11Ser	8.12E-06	26,23	99	0.167	0.001	1
6057	18	55315784	C	T	C/T	ATP8B1	Cholestasis, intrahepatic, of pregnancy, 1	c.3692G>A	p.Arg1231His	0	17,28	99	0.013	0.862	1
6057	17	59793346	G	C	G/C	BRIP1	Breast cancer	c.2458C>G	p.Gln820Glu	0	23,28	99	0	0.997	1
6057	16	70941377	C	T	C/T	HYDIN	Ciliary dyskinesia, primary, 5	c.8414G>A	p.Arg2805Gln	0.00031421	4,2	60	0.273	0.904	0.537
6058	22	51117070	C	T	C/T	SHANK3	Phelan-mcdermid syndrome	c.397C>T	p.Arg133Cys	1.63E-05	14,19	99	n/a	n/a	n/a
6058	11	77824929	T	A	T/A	ALG8	Congenital disorder of glycosylation, type Ih	c.777+3A>T		0	12,7	99	n/a	n/a	n/a
6058	11	77824928	T	A	T/A	ALG8	Congenital disorder of glycosylation, type Ih	c.777+4A>T		0	12,8	99	n/a	n/a	n/a
6058	2	220075562	C	T	C/T	ABCB6	Dyschromatosis universalis hereditaria 3	c.113G>A	p.Gly38Asp	4.09E-05	12,24	99	n/a	n/a	n/a

6058	1	156106126	C	T	C/T	LMNA	Hutchinson-gilford progeria syndrome	c.1279C>T	p.Arg427Cys	2.44E-05	29,22	99	0.041	0.017	1
6058	12	49434040	C	T	C/T	KMT2D	Kabuki syndrome 1	c.7513G>A	p.Glu2505Lys	0	15,17	99	0.019	0.009	0.898
6058	19	54656320	G	A	G/A	CNOT3	Neurodevelopmental disorder, CNOT3-related	c.1861G>A	p.Ala621Thr	2.03E-05	28,16	99	0.006	0.885	1
6058	10	88446933	A	G	A/G	LDB3	Myopathy, myofibrillar, 4	c.797A>G	p.His266Arg	3.23E-05	27,17	99	0.09	0.991	0.974
6058	3	127323591	G	A	G/A	MCM2	Deafness, autosomal dominant 70	c.377G>A	p.Arg126Gln	4.36E-05	29,18	99	0.096	0.007	0.807
6058	2	74590228	C	T	C/T	DCTN1	Perry syndrome	c.161G>A	p.Ser54Asn	1.22E-05	25,28	99	0.511	0.019	1
6060	X	69489509	T	G	T/G	ARR3	Myopia 26, X-linked, female-limited	c.9-3T>G		0	21,26	99	n/a	n/a	n/a
6060	12	49446073	A	G	A/G	KMT2D	Kabuki syndrome 1	c.1393T>C	p.Ser465Pro	0	19,22	99	0	0.55	1
6060	11	70333484	C	T	C/T	SHANK2	Autism, susceptibility to, 17	c.2917G>A	p.Gly973Ser	4.06E-06	17,19	99	0.368	0	1
6060	15	40468733	T	C	T/C	BUB1B	Colorectal cancer	c.482T>C	p.Ile161Thr	4.06E-05	24,28	99	0.077	1	1
6060	6	15651640	C	A	C/A	DTNBP1	Hermansky-pudlak syndrome 7	c.111-1G>T		4.56E-05	15,10	99	n/a	n/a	n/a
6060	X	119580228	G	A	G/A	LAMP2	Danon disease	c.796C>T	p.Arg266Cys	0	25,20	99	0.062	0.601	0.999
6060	2	48132788	CTGCTGT	C	CTGCTGT/C	FBXO11	Syndromic intellectual disability, FBXO11-related	c.66_71delACAGCA	p.Gln23_Gln24del	0	20,19	99	n/a	n/a	n/a
6060	1	6520127	C	T	C/T	ESPN	Deafness, autosomal recessive 36, with or without vestibular involvement	c.2486C>T	p.Thr829Met	1.50672E-04	20,19	99	0	0.999	0.685
6060	1	6508996	C	T	C/T	ESPN	Deafness, autosomal recessive 36, with or without vestibular involvement	c.1760C>T	p.Ala587Val	1.93E-05	24,16	99	0.081	0.03	1
6060	6	15651641	T	A	T/A	DTNBP1	Hermansky-pudlak syndrome 7	c.111-2A>T		4.15E-05	18,7	99	n/a	n/a	n/a
6060	7	2020107	C	CCCCCACAGGACACACCTGGGCGTGTCCGCCT	C/CCCCCACAGGACACACCTGGGCGTGTCCGCCT	MAD1L1	Prostate cancer	c.1485_1486insAGGCGGACACGCCCAGGTGTGTCCTGTGGGG	p.Glu496fs	0	27,13	99	n/a	n/a	n/a
6060	16	30976303	C	A	C/A	SETD1A	Schizophrenia, SETD1A-related	c.1240C>A	p.Pro414Thr	2.04E-05	19,22	99	0.002	0.172	0.537
6061	7	2946463	G	A	G/A	CARD11	B-cell expansion with NFKB and T-cell anergy	c.3274C>T	p.Arg1092*	2.05E-05	19,21	99	n/a	n/a	1
6061	19	13319575	G	A	G/A	CACNA1A	Migraine, familial hemiplegic, 1	c.6775C>T	p.Arg2259Trp	1.02E-05	19,18	99	0.001	0.982	0.998
6061	4	1808320	C	T	C/T	FGFR3	Achondroplasia	c.2084C>T	p.Ser695Phe	1.22E-05	18,18	99	0	0.968	1
6061	4	6279351	G	A	G/A	WFS1	Wolfram-like syndrome, autosomal dominant	c.169G>A	p.Ala57Thr	2.88E-05	30,22	99	0.354	0.001	1
6061	10	79814445	C	T	C/T	RPS24	Diamond-blackfan anemia 3	c.547C>T	p.Arg183Trp	2.02E-05	23,29	99	0	0	1
6061	1	158597431	TCTC	T	TCTC/T	SPTA1	Elliptocytosis 2	c.5645_5647delGAG	p.Gly1882del	2.85E-05	31,36	99	n/a	n/a	n/a

6061	11	77824929	T	A	T/A	ALG8	Congenital disorder of glycosylation, type Ih	c.777+3A>T		0	18,5	90	n/a	n/a	n/a
6061	11	77824928	T	A	T/A	ALG8	Congenital disorder of glycosylation, type Ih	c.777+4A>T		0	18,5	90	n/a	n/a	n/a
6061	19	42793352	C	T	C/T	CIC	Mental retardation, autosomal dominant 45	c.3881C>T	p.Ser1294Leu	1.23E-05	21,18	99	0	0.041	1
6061	17	5487135	G	A	G/A	NLRP1	Autoinflammation with arthritis and dyskeratosis	c.143C>T	p.Thr48Met	1.20982E-04	25,19	99	0.01	0.002	1
6061	2	228173993	C	T	C/T	COL4A3	Hematuria, benign familial	c.4714C>T	p.His1572Tyr	0	31,19	99	0.039	0.013	1
6061	1	110091422	G	C	G/C	GNAI3	Auriculocondylar syndrome 1	c.80G>C	p.Gly27Ala	0	20,27	99	0.317	0.018	1
6061	3	130174478	A	G	A/G	COL6A5	Familial neuropathic chronic itch, COL6A5-related	c.6758A>G	p.Gln2253Arg	0	21,39	99	0	0	1
6061	18	55328537	T	G	T/G	ATP8B1	Cholestasis, intrahepatic, of pregnancy, 1	c.2576A>C	p.Glu859Ala	0	23,16	99	0.743	0.987	1
6061	16	30976971	G	A	G/A	SETD1A	Schizophrenia, SETD1A-related	c.1769G>A	p.Arg590Gln	8.30E-06	20,20	99	0.007	0.005	0.938
6061	6	52344016	C	G	C/G	EFHC1	Epilepsy, myoclonic juvenile	c.1460C>G	p.Pro487Arg	0	23,24	99	0.004	0.998	1
6062	17	78078341	T	G	T/G	GAA	Glycogen storage disease ii	c.-32-13T>G		0.00347161	18,17	99	n/a	n/a	n/a
6062	6	152841678	C	CT	C/CT	SYNE1	Emery-dreifuss muscular dystrophy 4, autosomal dominant	c.226-2dupA		0.00012221	26,24	99	n/a	n/a	n/a
6062	17	78078882	C	T	C/T	GAA	Glycogen storage disease ii	c.497C>T	p.Thr166Ile	0	22,22	99	0	0.014	0.87
6062	20	61460844	G	C	G/C	COL9A3	Epiphyseal dysplasia, multiple, 3	c.1046G>C	p.Gly349Ala	2.86E-05	21,18	99	0	0.997	1
6062	9	140040289	G	A	G/A	GRIN1	Bilateral polymicrogyria, GRIN1-related	c.505G>A	p.Asp169Asn	8.28E-06	15,11	99	0.205	0.71	1
6062	8	41790648	T	G	T/G	KAT6A	Mental retardation, autosomal dominant 32	c.5090A>C	p.Gln1697Pro	2.01E-05	25,11	99	0.002	0	0.946
6062	7	151873293	G	C	G/C	KMT2C	Kleefstra syndrome 2	c.9245C>G	p.Pro3082Arg	2.08E-05	25,16	99	0.001	0.997	1
6062	3	58145335	G	A	G/A	FLNB	Larsen syndrome	c.7036G>A	p.Ala2346Thr	2.44E-05	15,18	99	0.019	0.754	1
6062	2	48030685	C	T	C/T	MSH6	Colorectal cancer, hereditary nonpolyposis, type 5	c.3299C>T	p.Thr1100Met	4.06E-05	16,17	99	0.024	0.105	1
6062	12	133218272	G	C	G/C	POLE	Colorectal cancer, susceptibility to, 12	c.5339C>G	p.Ala1780Gly	2.44E-05	16,18	99	0.12	0.033	1
6062	17	40948262	C	T	C/T	WNK4	Pseudohypoaldosteronism, type iib	c.3553C>T	p.Arg1185Cys	1.49E-04	19,23	99	0	0.997	1
6064	5	122491651	A	G	A/G	PRDM6	Patent ductus arteriosus 3	c.974A>G	p.Tyr325Cys	0	23,31	99	0	0.999	1
6064	5	37024753	A	G	A/G	NIPBL	Cornelia de Lange syndrome 1	c.5641A>G	p.Thr1881Ala	8.14E-06	36,30	99	0.535	0.011	0.998
6064	4	5633750	C	G	C/G	EVC2	Ellis-van creveld syndrome	c.1480G>C	p.Glu494Gln	8.12E-06	28,20	99	0.127	0.999	0.998
6064	7	100776994	A	G	A/G	SERPINE1	Plasminogen activator inhibitor-1 deficiency	c.719A>G	p.Asp240Gly	1.22E-05	23,20	99	0.071	0.004	1

6064	4	114278533	A	G	A/G	ANK2	Cardiac arrhythmia, ankyrin-b-related	c.8759A>G	p.Tyr2920Cys	8.14E-06	30,31	99	0.063	0	1
6064	20	31022679	C	T	C/T	ASXL1	Bohring-opitz syndrome	c.2164C>T	p.Pro722Ser	0	29,22	99	0	0	1
6064	16	30748820	C	A	C/A	SRCAP	Floating-harbor syndrome	c.7459C>A	p.Pro2487Thr	8.35E-06	19,26	99	0.009	0.064	1
6064	4	5576420	A	G	A/G	EVC2	Ellis-van creveld syndrome	c.3352T>C	p.Cys1118Arg	0	34,13	99	0.033	0.184	0.975
6065	15	48780436	T	C	T/C	FBN1	Marfan syndrome	c.3211A>G	p.Ile1071Val	4.06E-06	29,23	99	0.051	0.97	1
6065	7	151874064	T	G	T/G	KMT2C	Kleefstra syndrome 2	c.8474A>C	p.Glu2825Ala	4.07E-06	23,17	99	0.003	0.265	0.998
6065	2	8871889	GA	G	GA/G	KIDINS220	Spastic paraplegia, intellectual disability, nystagmus, and obesity	c.4276delT	p.Ser1426fs	0	22,28	99	n/a	n/a	n/a
6065	5	176829295	A	G	A/G	F12	Angioedema, hereditary, type iii	c.1846T>C	p.Ter616Argext*?	8.15E-06	21,21	99	n/a	n/a	1
6065	19	15292406	G	C	G/C	NOTCH3	Lateral meningocele syndrome	c.2773C>G	p.Leu925Val	3.44E-05	24,19	99	1	0.005	0.903
6065	3	47859602	C	T	C/T	DHX30	Neurodevelopmental disorder with severe motor impairment and absent language	c.119C>T	p.Ser40Phe	4.11E-06	26,20	99	0.025	737	1
6066	X	13769399	A	G	A/G	OFD1	Orofaciodigital syndrome I	c.967A>G	p.Ser323Gly	5.62E-06	26,20	99	0.009	0.242	1
6066	3	119120823	G	T	G/T	ARHGAP31	Adams-Oliver syndrome 1	c.1224G>T	p.Glu408Asp	0	15,14	99	0.006	0.039	0.507
6066	1	27023087	C	T	C/T	ARID1A	Coffin-siris syndrome 2	c.193C>T	p.Pro65Ser	0	21,20	99	0	0.007	0.823
6066	5	176831047	C	G	C/G	F12	Angioedema, hereditary, type iii	c.1063G>C	p.Gly355Arg	8.86E-06	16,18	99	0.03	0.002	1
6066	3	127318385	G	A	G/A	MCM2	Deafness, autosomal dominant 70	c.231G>A	p.Met77Ile	2.87E-05	24,15	99	0.041	0.266	1
6068	1	45292158	A	G	A/G	PTCH2	Basal cell nevus syndrome	c.2976+2T>C		3.68E-05	15,12	99	n/a	n/a	1
6068	9	135772901	G	A	G/A	TSC1	Tuberous sclerosis 1	c.2722C>T	p.Arg908Trp	2.03E-05	11,21	99	0	0.999	0.999
6068	2	179537361	C	T	C/T	TTN	Tibial muscular dystrophy, tardive	c.34855+1G>A		4.55E-05	20,32	99	n/a	n/a	1
6068	1	158584070	GTC	G	GTC/G	SPTA1	Elliptocytosis 2	c.6813_6814delGA	p.Glu2271fs	0	26,24	99	n/a	n/a	n/a
6068	19	36209038	G	A	G/A	KMT2B	Dystonia 28, childhood-onset	c.118G>A	p.Ala40Thr	0	8,14	99	0	0.676	0.844
6068	9	133760552	G	A	G/A	ABL1	Congenital heart defects and skeletal malformations syndrome	c.2932G>A	p.Val978Met	3.68E-05	16,19	99	0.089	0.075	1
6068	16	56868350	G	A	G/A	NUP93	Nephrotic syndrome, type 12	c.1733G>A	p.Arg578Gln	6.09E-05	10,9	99	0.004	0.046	1
6068	16	56867190	C	T	C/T	NUP93	Nephrotic syndrome, type 12	c.1409C>T	p.Ala470Val	4.36892E-04	18,23	99	0.126	0.256	1
6068	13	100637720	GGCGGCGGCT	G	GGCGGCGGCT/G	ZIC2	Holoprosencephaly 5	c.1392_1400delTGCGGCGGC	p.Ala465_Ala467del	1.49E-05	8,11	99	n/a	n/a	n/a
6068	6	73108734	C	T	C/T	RIMS1	Cone-rod dystrophy 7	c.4798C>T	p.Leu1600Phe	4.88E-05	20,25	99	0.002	0.638	0.946

6068	3	129290053	G	A	G/A	PLXND1	Moebius syndrome, PLXND1-related	c.3430C>T	p.Arg1144Trp	4.87E-05	14,19	99	0.07	0.663	0.636
6068	5	74021509	A	T	T/T	GFM2	Mitochondrial disease, GFM2-related	c.1984T>A	p.Ser662Thr	5.34001E-04	0,33	99	0.13	0.646	1
6068	9	5089702	G	A	G/A	JAK2	Myelofibrosis	c.2600G>A	p.Arg867Gln	5.52E-06	13,18	99	0.108	0.916	1
6068	2	179571408	C	T	C/T	TTN	Tibial muscular dystrophy, tardive	c.29193G>A	p.Trp9731*	0	25,19	99	n/a	n/a	1
6068	5	11385275	G	C	G/C	CTNND2	Familial cortical myoclonic tremor and epilepsy (FCMTE), CTNND2-related	c.679C>G	p.Arg227Gly	4.09E-05	10,19	99	0.001	0	0.641
6068	2	179418917	C	T	C/T	TTN	Tibial muscular dystrophy, tardive	c.88921G>A	p.Glu29641Lys	1.28E-05	21,15	99	0.039	0.064	1
6069	16	3293405	C	T	C/T	MEFV	Familial mediterranean fever, autosomal dominant	c.2082G>A	p.Met694Ile	0.00013806	24,15	99	0.113	0.337	0
6069	17	17700322	G	A	G/A	RAI1	Smith-magenis syndrome	c.4060G>A	p.Gly1354Ser	4.06E-06	20,19	99	0.025	0.607	1
6069	5	90119286	A	C	A/C	ADGRV1	Febrile seizures, familial, 4	c.16241A>C	p.Lys5414Thr	4.06E-06	28,18	99	0.1	0.021	0.991
6069	5	89923109	A	G	A/G	ADGRV1	Febrile seizures, familial, 4	c.754A>G	p.Ile252Val	1.22E-05	26,20	99	1	0.001	0.976
6069	20	57598966	C	T	C/T	TUBB1	Macrothrombocytopenia, autosomal dominant, tubb1-related	c.484C>T	p.Arg162Trp	4.06E-05	18,13	99	0	1	1
6069	3	168845656	T	A	T/A	MECOM	Radioulnar synostosis with amegakaryocytic thrombocytopenia 2	c.806A>T	p.Asp269Val	4.07E-06	15,26	99	0.012	0.976	1
6069	6	100838484	T	G	T/G	SIM1	Obesity	c.2054A>C	p.His685Pro	4.06E-06	22,22	99	0.038	0.001	0.999
6069	3	126723457	A	T	A/T	PLXNA1	Developmental encephalopathy, PLXNA1-related	c.1522A>T	p.Thr508Ser	5.71E-06	21,26	99	1	0.001	1
6069	17	39023071	C	G	C/G	KRT12	Corneal dystrophy, meesmann	c.368G>C	p.Gly123Ala	8.12E-06	17,22	99	0.021	0.193	0.995
6070	7	103234153	T	A	T/A	RELN	Epilepsy, familial temporal lobe, 7	c.3888A>T	p.Lys1296Asn	4.07E-06	21,18	99	0.207	0.5	0.75
6070	6	33399977	G	C	G/C	SYNGAP1	Mental retardation, autosomal dominant 5	c.335G>C	p.Gly112Ala	1.22E-05	15,20	99	0	0.054	0.95
6070	11	57369597	A	T	A/T	SERPING1	Angioedema, hereditary, type i	c.742A>T	p.Thr248Ser	2.84E-05	12,12	99	0.825	0	1
6070	13	101710354	G	A	G/A	NALCN	Congenital contractures of the limbs and face, hypotonia, and developmental delay	c.4960C>T	p.Arg1654Trp	0	19,15	99	0.008	0.336	1
6070	8	41805286	C	T	C/T	KAT6A	Mental retardation, autosomal dominant 32	c.1885G>A	p.Val629Ile	1.78E-05	20,20	99	0.028	0.431	1
6070	7	156802369	T	C	T/C	MNX1	Currarino syndrome	c.676A>G	p.Met226Val	1.74E-05	23,27	99	0.051	0.018	0.943
6071	8	38162273	G	A	G/A	NSD3	Leukemia, acute myeloid	c.2443C>T	p.Arg815Cys	4.28E-05	20,16	99	0	0.996	1
6071	1	196654231	C	G	C/G	CFH	Hemolytic uremic syndrome, atypical, susceptibility to, 1	c.828C>G	p.Asp276Glu	4.07E-06	24,20	99	0.274	0.358	1

6071	13	100634398	C	T	C/T	ZIC2	Holoprosencephaly 5	c.80C>T	p.Ala27Val	3.26E-05	15,22	99	0.05	0.75	0.842
6071	17	41256210	T	G	T/G	BRCA1	Breast-ovarian cancer, familial, susceptibility to, 1	c.370A>C	p.Ile124Leu	0	20,28	99	0.069	0.132	1
6071	9	133589732	T	C	T/C	ABL1	Congenital heart defects and skeletal malformations syndrome	c.26T>C	p.Leu9Pro	8.15E-06	18,17	99	0.1	0.003	1
6071	17	79478801	G	A	G/A	ACTG1	Baraitser-winter syndrome 2	c.392C>T	p.Pro131Leu	2.38E-05	14,17	99	n/a	n/a	n/a
6071	11	18505518	G	A	G/A	TSG101	Breast cancer	c.742C>T	p.Arg248Cys	2.03E-05	26,18	99	0.01	0.326	1
6072	14	94780642	A	T	A/T	SERPINA6	Corticosteroid-binding globulin deficiency	c.344T>A	p.Leu115His	0.00224263	24,12	99	0.026	0.702	0.73
6072	12	52312953	T	A	T/A	ACVRL1	Telangiectasia, hereditary hemorrhagic, type 2	c.435T>A	p.Asp145Glu	1.23E-05	17,15	99	n/a	n/a	n/a
6072	12	133256632	C	A	C/A	POLE	Colorectal cancer, susceptibility to, 12	c.331G>T	p.Gly111Cys	4.07E-06	24,24	99	0.003	0.94	1
6072	1	169495249	A	C	A/C	F5	Thrombophilia due to activated protein c resistance	c.5621T>G	p.Phe1874Cys	8.15E-06	21,18	99	0.002	0.982	1
6072	3	98311934	C	G	C/G	CPOX	Coproporphyria, hereditary	c.415G>C	p.Gly139Arg	3.69E-05	12,15	99	0.795	0.042	1
6072	20	60942192	T	TCCCGCG	T/TCCCGCG	LAMA5	Extracellular matrix syndrome, LAMA5-related	c.104_109dupCGCGGG	p.Ala35_Arg36dup	3.39E-05	25,16	99	n/a	n/a	n/a
6072	20	60887333	G	A	G/A	LAMA5	Extracellular matrix syndrome, LAMA5-related	c.9400C>T	p.Arg3134Cys	3.76E-05	15,27	99	0.067	0.132	0.997
6072	1	35250486	G	T	G/T	GJB3	Erythrokeratodermia variabilis et progressiva 1	c.123G>T	p.Glu41Asp	4.87E-05	13,9	99	0.003	0.983	0.992
6072	1	182555434	T	C	T/C	RNASEL	Prostate cancer, hereditary, 1	c.508A>G	p.Thr170Ala	1.63E-05	17,19	99	0.073	0.998	0.98
6072	12	1017692	A	G	A/G	WNK1	Pseudohypoaldosteronism, type iic	c.8377A>G	p.Met2793Val	4.06E-06	16,21	99	0.012	0.216	0.999
6072	7	151945330	G	T	G/T	KMT2C	Kleefstra syndrome 2	c.2189C>A	p.Ser730Tyr	4.15E-05	50,13	99	0.017	0.185	0.999
6072	1	152276467	T	TGGA	T/TGGA	FLG	Ichthyosis vulgaris	c.10894_10895insTCC	p.Gln3631_Gln3632insLeu	4.08E-06	16,4	99	n/a	n/a	n/a
6072	1	152276444	A	C	A/C	FLG	Ichthyosis vulgaris	c.10918T>G	p.Ser3640Ala	2.47E-05	19,6	99	0.182	0.948	1
6073	4	79321971	A	C	A/C	FRAS1	Fraser syndrome 1	c.4059A>C	p.Leu1353Phe	2.44E-05	21,25	99	0	0.999	1
6073	4	79301101	G	A	G/A	FRAS1	Fraser syndrome 1	c.3514G>A	p.Asp1172Asn	1.58685E-04	25,25	99	0.006	0.821	1
6073	2	98851159	G	T	G/T	VWA3B	Spinocerebellar ataxia, autosomal recessive 22	c.2357G>T	p.Ser786Ile	0	13,23	99	0.049	0.049	0.988
6073	2	98779328	G	A	G/A	VWA3B	Spinocerebellar ataxia, autosomal recessive 22	c.1003G>A	p.Glu335Lys	1.22E-05	18,21	99	0.071	0.79	0.581
6073	1	171605222	TA	T	TA/T	MYOC	Glaucoma 1, open angle, a	c.1357delT	p.Tyr453fs	4.87E-05	25,21	99	n/a	n/a	n/a
6073	2	32463363	G	C	G/C	NLRC4	Autoinflammation with infantile enterocolitis	c.2359C>G	p.Leu787Val	4.15E-06	22,18	99	0.08	0.994	n/a
6073	10	64572977	C	T	C/T	EGR2	Hypertrophic neuropathy of dejerine-sottas	c.1421G>A	p.Arg474Gln	1.47E-05	23,19	99	0	0.721	0.943

6073	19	39062819	G	A	G/A	RYR1	Central core disease of muscle	c.13907G>A	p.Ser4636Asn	4.06E-06	21,32	99	0.362	0.986	1
6073	2	179615375	C	T	C/T	TTN	Lethal congenital contracture syndrome, TTN-related	c.11752G>A	p.Glu3918Lys	2.45E-05	18,30	99	0.024	0.004	1
6073	2	179611689	C	G	C/G	TTN	Lethal congenital contracture syndrome, TTN-related	c.15438G>C	p.Glu5146Asp	1.22E-05	26,31	99	0	0.996	1
6073	2	179415783	T	C	T/C	TTN	Lethal congenital contracture syndrome, TTN-related	c.91475A>G	p.Tyr30492Cys	2.04E-05	23,31	99	0.001	0.905	0.999
6073	18	24081207	C	T	C/T	KCTD1	Scalp-ear-nipple syndrome	c.1817G>A	p.Arg606Gln	0	31,30	99	n/a	n/a	1
6073	17	79801469	G	A	G/A	P4HB	Cole-Carpenter syndrome 1	c.241C>T	p.Arg81Trp	3.23E-05	16,27	99	n/a	n/a	1
6073	17	78362464	T	C	T/C	RNF213	Moyamoya disease 2	c.15122T>C	p.Met5041Thr	0	21,15	99	0.246	0.003	1
6073	16	1270819	C	T	C/T	CACNA1H	Hyperaldosteronism, familial, type iv	c.6887C>T	p.Ser2296Phe	1.22E-05	20,30	99	0.149	0.001	1
6073	14	65544579	G	A	G/A	MAX	Pheochromocytoma	c.347C>T	p.Ser116Leu	0	15,18	99	0.19	0	1
6073	10	70191971	A	G	A/G	DNA2	Progressive external ophthalmoplegia with mitochondrial DNA deletions, autosomal dominant 6	c.2123T>C	p.Ile708Thr	4.01E-05	28,34	99	0.018	0.999	1
6073	8	103250875	C	T	C/T	RRM2B	Progressive external ophthalmoplegia with mitochondrial dna deletions, autosomal dominant 5	c.128G>A	p.Arg43Gln	2.63816E-04	17,18	99	n/a	n/a	n/a
6073	3	129249826	G	A	G/A	RHO	Retinitis pigmentosa 4	c.469G>A	p.Val157Ile	1.34015E-04	20,24	99	0.238	0.006	1
6073	3	48623783	G	A	G/A	COL7A1	Epidermolysis bullosa dystrophica, autosomal dominant	c.3532C>T	p.Arg1178Cys	4.06E-05	27,16	99	0.001	0.963	1
6073	1	152281949	C	T	C/T	FLG	Ichthyosis vulgaris	c.5413G>A	p.Ala1805Thr	3.25E-05	19,13	99	0.374	0.971	1
6073	1	40770189	C	T	C/T	COL9A2	Epiphyseal dysplasia, multiple, 2	c.1174G>A	p.Glu392Lys	3.23E-05	15,29	99	0.018	0.766	1
6073	9	124064254	T	C	T/C	GSN	Amyloidosis, finnish type	c.158T>C	p.Val53Ala	0	13,15	99	0.082	0.003	0.736
6074	11	17491699	C	T	C/T	ABCC8	Diabetes mellitus, permanent neonatal	c.361G>A	p.Val121Met	0	14,32	99	0.01	0.657	0.934
6074	2	179460232	A	T	A/T	TTN	Lethal congenital contracture syndrome, TTN-related	c.57847+2T>A		0.00066746	27,7	99	n/a	n/a	1
6074	12	65564118	C	G	C/G	LEMD3	Buschke-ollendorff syndrome	c.742C>G	p.Arg248Gly	0	26,16	99	0.001	0.002	0.857
6074	16	88788122	C	G	C/G	PIEZO1	Dehydrated hereditary stomatocytosis 1 with or without pseudohyperkalemia and/or perinatal edema	c.5227G>C	p.Val1743Leu	2.21E-05	20,22	99	0.028	0.058	0.913
6074	9	139396470	C	G	C/G	NOTCH1	Adams-Oliver syndrome 5	c.5455G>C	p.Glu1819Gln	0	22,18	99	0.463	0.354	1

6074	2	179669299	G	T	G/T	TTN	Lethal congenital contracture syndrome, TTN-related	c.71C>A	p.Thr24Asn	1.22E-05	17,33	99	0.152	0.997	1
6074	2	179479607	G	T	G/T	TTN	Lethal congenital contracture syndrome, TTN-related	c.48727C>A	p.Pro16243Thr	3.16E-05	24,20	99	0.2	0.253	0.999
6074	2	179393395	T	C	T/C	TTN	Lethal congenital contracture syndrome, TTN-related	c.107083A>G	p.Ser35695Gly	1.64E-05	32,16	99	0	0.133	1
6074	1	152284996	C	A	C/A	FLG	Ichthyosis vulgaris	c.2366G>T	p.Arg789Leu	4.06E-06	19,18	99	0.02	0.034	1
6074	6	16328348	G	T	G/T	ATXN1	Spinocerebellar ataxia 1	c.194C>A	p.Thr65Asn	4.31E-06	17,24	99	0.072	0.092	0.927
6074	5	126113223	C	A	C/A	LMNB1	Leukodystrophy, demyelinating, adult-onset, autosomal dominant	c.23C>A	p.Pro8Gln	0	28,19	99	0.31	0.048	0.934
6075	7	151328758	A	G	A/G	PRKAG2	Glycogen storage disease of heart, lethal congenital	c.128T>C	p.Leu43Pro	3.86E-05	14,14	99	n/a	n/a	n/a
6075	2	179474019	G	A	G/A	TTN	Salih myopathy	c.52018C>T	p.Leu17340Phe	1.03241E-04	18,14	99	0.204	0.095	1
6075	2	179396631	C	T	C/T	TTN	Salih myopathy	c.104711G>A	p.Arg34904Lys	9.88E-05	21,29	99	0.154	0.031	0.992
6075	12	115112440	C	T	C/T	TBX3	Ulnar-mammary syndrome	c.1300G>A	p.Asp434Asn	1.77E-05	12,18	99	0.002	0.808	1
6075	7	87038632	T	C	T/C	ABCB4	Gallbladder disease 1	c.3001A>G	p.Lys1001Glu	8.13E-06	14,15	99	0.002	0.297	1
6076	1	116206792	G	A	G/A	VANGL1	Sacral defect with anterior meningocele	c.715G>A	p.Val239Ile	4.89E-05	17,18	99	0.352	0.017	1
6076	11	61546324	C	T	C/T	MYRF	Cardiac and urogenital anomalies syndrome, MYRF-related	c.940C>T	p.Pro314Ser	0	22,23	99	n/a	n/a	n/a
6076	22	29108001	C	G	C/G	CHEK2	Li-fraumeni syndrome 2	c.817G>C	p.Ala273Pro	4.06E-06	29,25	99	0.004	0.978	1
6076	13	32944659	G	A	G/A	BRCA2	Breast-ovarian cancer, familial, susceptibility to, 2	c.8452G>A	p.Val2818Ile	4.06E-06	26,25	99	0.192	0.053	0.944
6076	22	51159250	C	G	C/G	SHANK3	Phelan-mcdermid syndrome	c.3037C>G	p.Leu1013Val	3.91E-05	18,9	99	n/a	n/a	n/a
6076	11	57367583	A	C	A/C	SERPING1	Angioedema, hereditary, type i	c.385A>C	p.Thr129Pro	4.07E-06	16,5	85	0.254	0.029	1
6076	18	58039494	G	A	G/A	MC4R	Obesity	c.89C>T	p.Ser30Phe	8.53E-05	22,25	99	0.069	0.005	0.998
6076	11	77824929	T	A	T/A	ALG8	Congenital disorder of glycosylation, type Ih	c.777+3A>T		0	13,4	83	n/a	n/a	n/a
6076	11	77824928	T	A	T/A	ALG8	Congenital disorder of glycosylation, type Ih	c.777+4A>T		0	13,4	83	n/a	n/a	n/a
6076	21	38884369	T	TCAC	T/TCAC	DYRK1A	Mental retardation, autosomal dominant 7	c.1842_1844dupCCA	p.His615dup	1.63E-05	18,20	99	n/a	n/a	n/a
6076	14	65260322	G	A	G/A	SPTB	Spherocytosis, type 2	c.2059C>T	p.Arg687Cys	2.44E-05	23,12	99	0.017	0.935	0.923
6076	18	55322586	T	C	T/C	ATP8B1	Cholestasis, intrahepatic, of pregnancy, 1	c.2771A>G	p.Tyr924Cys	3.25E-05	28,21	99	0	0.603	1
6076	3	38888502	A	G	A/G	SCN11A	Episodic pain syndrome, familial, 3	c.5059T>C	p.Phe1687Leu	4.07E-06	21,8	99	0.118	0.049	0.992

6077	3	45801497	T	C	T/C	SLC6A20	Hyperglycinuria	c.1481A>G	p.Lys494Arg	4.13E-06	22,7	99	0.164	0.001	0.984
6077	12	40619004	A	G	A/G	LRRK2	Parkinson disease 8, autosomal dominant	c.71A>G	p.Asn24Ser	1.22E-05	21,23	99	0.033	0.014	0.882
6077	8	41513269	G	T	G/T	ANK1	Spherocytosis, type 1	c.5623C>A	p.His1875Asn	1.22E-05	27,11	99	0.202	0.922	0.613
6077	2	238289629	C	T	C/T	COL6A3	Ullrich congenital muscular dystrophy 1	c.1826G>A	p.Arg609Gln	0.00015847	17,25	99	0.048	0.778	0.565
6077	7	151876959	G	A	G/A	KMT2C	Kleefstra syndrome 2	c.7402C>T	p.Pro2468Ser	4.88E-05	13,13	99	0.182	0.018	0.991
6077	17	38225262	T	C	T/C	THRA	Hypothyroidism, congenital, nongoitrous, 6	c.-298+2T>C		0	5,13	99	n/a	n/a	n/a
6077	5	180050979	C	T	C/T	FLT4	Lymphedema, hereditary, ia	c.1504G>A	p.Glu502Lys	2.03E-05	13,25	99	0.005	0.999	1
6077	12	14834386	C	T	C/T	GUCY2C	Diarrhea 6	c.637G>A	p.Val213Ile	3.25E-05	29,29	99	0.442	0.015	0.954
6080	11	5647830	C	T	C/T	HBG2	Fetal hemoglobin quantitative trait locus 1	c.-883+1G>A		0	17,22	99	n/a	n/a	n/a
6080	18	42532509	G	T	G/T	SETBP1	Schinzel-giedion midface retraction syndrome	c.3204G>T	p.Gln1068His	0	19,26	99	0.01	0.855	0.612
6080	16	2140691	G	A	G/A	PKD1	Polycystic kidney disease 1	c.12122C>T	p.Ala4041Val	0	16,25	99	0.129	0.03	0.995
6080	11	77835184	T	C	T/C	ALG8	Congenital disorder of glycosylation, type Ih	c.251A>G	p.Tyr84Cys	0.00028021	36,31	99	0.057	0.053	0.982
6080	11	77818809	A	T	A/T	ALG8	Congenital disorder of glycosylation, type Ih	c.494T>A	p.Val165Asp	3.99007E-04	18,17	99	0.034	n/a	1
6080	3	52407015	G	A	G/A	DNAH1	Ciliary dyskinesia, primary, 37	c.6931G>A	p.Glu2311Lys	8.14E-06	22,23	99	0	1	1
6080	19	15300156	G	A	G/A	NOTCH3	Lateral meningocele syndrome	c.1120C>T	p.Arg374Trp	0	30,21	99	0.004	0.983	1
6080	3	52378564	A	G	A/G	DNAH1	Ciliary dyskinesia, primary, 37	c.1345A>G	p.Met449Val	8.12E-06	28,22	99	0.058	0.053	1
6080	2	179440042	A	G	A/G	TTN	Lethal congenital contracture syndrome, TTN-related	c.70817T>C	p.Met23606Thr	4.48E-05	18,35	99	0.19	0.233	1
6080	2	179415885	C	T	C/T	TTN	Lethal congenital contracture syndrome, TTN-related	c.91373G>A	p.Ser30458Asn	7.74E-05	27,29	99	0.453	0.031	0.995
6080	2	179397349	G	C	G/C	TTN	Lethal congenital contracture syndrome, TTN-related	c.103993C>G	p.Leu34665Val	8.13E-05	28,25	99	0.024	0.035	0.992
6080	X	32536177	A	G	A/G	DMD	Cardiomyopathy, dilated, 3b	c.2240T>C	p.Phe747Ser	0	34,40	99	0.111	0.994	0.995
6080	X	22196493	A	T	A/T	PHEX	Hypophosphatemic rickets, X-linked dominant	c.1586A>T	p.Glu529Val	0	31,35	99	0.719	0.669	1
6080	9	98221963	C	A	C/A	PTCH1	Basal cell nevus syndrome	c.2806G>T	p.Ala936Ser	0	26,35	99	0.022	0.965	1
6080	2	121746723	TGGACGA	T	TGGACGA/T	GLI2	Holoprosencephaly 9	c.3236_3241delACGAGG	p.Asp1079_Glu1080del	0	20,28	99	n/a	n/a	n/a
6080	1	3342786	A	G	A/G	PRDM16	Left ventricular noncompaction 8 / Cardiomyopathy, dilated,	c.3281A>G	p.Lys1094Arg	4.07E-06	35,22	99	0.388	0.395	0.999

							1LL								
6081	1	196658682	G	A	G/A	CFH	Hemolytic uremic syndrome, atypical, susceptibility to, 1	c.1097G>A	p.Gly366Glu	2.04E-05	28,36	99	0.689	1	1
6081	12	133252406	C	G	C/G	POLE	Colorectal cancer, susceptibility to, 12	c.1021G>C	p.Ala341Pro	0	22,12	99	0	0.677	1
6081	2	220285590	C	A	C/A	DES	Myopathy, myofibrillar, 1	c.938C>A	p.Ala313Asp	0	20,14	99	0.001	0.938	1
6081	19	36212117	CAG	C	CAG/C	KMT2B	Dystonia 28, childhood-onset	c.1869_1870delAG	p.Ala624fs	4.69E-05	8,3	96	n/a	n/a	n/a
6081	2	179649068	G	T	G/T	TTN	Lethal congenital contracture syndrome, TTN-related	c.2504C>A	p.Ala835Asp	3.68E-05	25,27	99	0	0.873	0.994
6081	2	179659795	A	G	A/G	TTN	Lethal congenital contracture syndrome, TTN-related	c.1099T>C	p.Ser367Pro	1.22E-05	27,15	99	0.021	0.073	1
6081	17	48694902	C	T	C/T	CACNA1G	Spinocerebellar ataxia 42	c.5125C>T	p.Arg1709Cys	0	22,24	99	0	0.992	1
6081	1	1167780	C	T	C/T	B3GALT6	Spondyloepimetaphyseal dysplasia with joint laxity, type 1, with or without fractures	c.122C>T	p.Ala41Val	3.98E-05	16,21	99	0.292	0.008	1
6081	1	1168608	C	T	C/T	B3GALT6	Spondyloepimetaphyseal dysplasia with joint laxity, type 1, with or without fractures	c.950C>T	p.Pro317Leu	0	34,20	99	0.149	0.236	1

Supplementary Table 2: The number of HPO terms extracted from patient EHRs at enrollment and reanalysis. ***** indicates patient mortality between the time of enrollment and reanalysis; **a** indicates prior publication in Farnaes et al; **b** indicates prior publication in Sanford et al.

Family ID	Number of HPO terms at enrollment	Number of HPO terms at reanalysis	% increase in number of phenotypes	Months elapsed between enrollment and reanalysis
6001*[a]	405	678	67.4	23
6003[a]	74	120	62.2	23
6005[a]	28	39	39.3	23
6006[b]	102	297	191.2	23
6009[a]	250	514	105.6	22
6010	86	158	83.7	22
6013	334	528	58.1	22
6015*	493	558	13.2	21
6016	287	415	44.6	21
6017[a]	101	295	192.1	21
6022[a]	162	261	61.1	21
6023*[a]	71	132	85.9	21
6025	182	478	162.6	21
6027[a]	162	285	75.9	20
6032	95	297	212.6	20
6033	460	679	47.6	19
6034*[a]	162	247	52.5	20
6035	157	291	85.4	20
6036*[a]	91	104	14.3	19
6037[a]	66	173	162.1	19
6038[a]	271	442	63.1	19
6040[a]	149	203	36.2	19
6043*	266	311	16.9	20
6046*[a]	42	63	50.0	19
6047[a]	157	470	199.4	19
6049*[a]	316	497	57.3	19
6050	212	265	25.0	19
6055	501	606	21.0	18
6057	133	265	99.2	18
6058	177	217	22.6	18
6060[a]	213	244	14.6	18

6061	210	242	15.2	18
6062	371	387	4.3	18
6064[a]	197	268	36.0	18
6065*[a]	140	169	20.7	18
6066[a]	149	176	18.1	18
6068	403	452	12.2	17
6069[a]	40	181	352.5	17
6070[a]	52	59	13.5	17
6071[a]	145	198	36.6	17
6072*[a]	291	297	2.1	17
6073[b]	147	268	82.3	17
6074	88	196	122.7	17
6075	93	113	21.5	17
6076	231	420	81.8	17
6077	80	148	85.0	17
6080	227	283	24.7	16
6081	47	195	314.9	16
Median	*160*	*267*	*55*	*19*
1st Quartile	*93*	*180*	*20*	*17*
3rd Quartile	*254*	*416*	*85*	*21*
Min	*28*	*39*	*2*	*16*
Max	*501*	*679*	*353*	*23*

Supplementary Table 3: Representation of HPO term subcategories in the extracted HPO term lists. Y: a term falling within this subcategory was present on the HPO term list; N: no terms falling within this subcategory were present on the HPO term list.

	Number of categories	Difference	HP:0000119-Abnormality of the genitourinary system	HP:0000152-Abnormality of head or neck	HP:0000478-Abnormality of the eye	HP:0000598-Abnormality of the ear	HP:0000707-Abnormality of the nervous system	HP:0000769-Abnormality of the breast	HP:0000818-Abnormality of the endocrine system	HP:0000924-Abnormality of the skeletal system	HP:0001197-Abnormality of prenatal development or birth	HP:0001507-Growth abnormality	HP:0001574-Abnormality of the integument	HP:0001608-Abnormality of the voice	HP:0001626-Abnormality of the cardiovascular system	HP:0001871-Abnormality of blood and blood-forming tissues	HP:0001939-Abnormality of metabolism/homeostasis	HP:0002086-Abnormality of the respiratory system	HP:0002664-Neoplasm	HP:0002715-Abnormality of the immune system	HP:0003011-Abnormality of the musculature	HP:0003549-Abnormality of connective tissue	HP:0025031-Abnormality of the digestive system	HP:0025142-Constitutional symptom	HP:0025354-Abnormal cellular phenotype	HP:0040064-Abnormality of limbs	HP:0045027-Abnormality of the thoracic cavity	HP:0500014-Abnormal test result
6001-Enrollment	23	0	Y	Y	Y	Y	Y	N	Y	Y	Y	Y	Y	Y	Y	Y	Y	Y	Y	Y	Y	Y	Y	Y	N	Y	Y	N
6001-Reanalysis	23		Y	Y	Y	Y	Y	N	Y	Y	Y	Y	Y	Y	Y	Y	Y	Y	Y	Y	Y	Y	Y	Y	N	Y	Y	N
6003-Enrollment	13	3	N	Y	Y	N	Y	N	N	N	N	N	Y	Y	Y	Y	Y	Y	N	Y	Y	N	Y	Y	N	N	N	N
6003-Reanalysis	16		N	Y	Y	N	Y	N	N	Y	N	Y	Y	Y	Y	Y	Y	Y	N	Y	Y	N	Y	Y	N	Y	N	N
6005-Enrollment	9	2	N	Y	Y	N	N	N	N	N	Y	N	Y	N	Y	Y	Y	N	N	Y	N	N	Y	N	N	N	N	N
6005-Reanalysis	11		N	Y	Y	N	Y	N	N	N	Y	Y	Y	N	Y	Y	Y	N	N	Y	N	N	Y	N	N	N	N	N
6006-Enrollment	14	7	N	Y	Y	N	Y	N	N	Y	N	N	Y	N	Y	N	Y	Y	N	Y	Y	Y	Y	Y	N	Y	N	N
6006-Reanalysis	21		Y	Y	Y	Y	Y	N	N	Y	Y	N	Y	Y	Y	Y	Y	Y	Y	Y	Y	Y	Y	Y	N	Y	Y	N
6009-Enrollment	19	5	Y	Y	Y	N	Y	N	N	Y	Y	Y	Y	N	Y	Y	Y	Y	Y	Y	N	Y	Y	Y	N	Y	Y	N
6009-Reanalysis	24		Y	Y	Y	Y	Y	N	Y	Y	Y	Y	Y	Y	Y	Y	Y	Y	Y	Y	Y	Y	Y	Y	N	Y	Y	Y
6010-Enrollment	14	4	N	Y	N	N	Y	N	N	Y	N	N	Y	N	Y	Y	Y	Y	N	Y	N	N	Y	Y	N	Y	Y	Y
6010-Reanalysis	18		N	Y	Y	Y	Y	N	N	Y	N	N	Y	N	Y	Y	Y	Y	N	Y	Y	Y	Y	Y	N	Y	Y	Y
6013-Enrollment	23	0	Y	Y	Y	Y	Y	N	Y	Y	Y	Y	Y	Y	Y	Y	Y	Y	Y	Y	Y	Y	Y	Y	N	Y	Y	N
6013-Reanalysis	23		Y	Y	Y	Y	Y	N	Y	Y	Y	Y	Y	Y	Y	Y	Y	Y	Y	Y	Y	Y	Y	Y	N	Y	Y	N
6015-Enrollment	25	0	Y	Y	Y	Y	Y	Y	Y	Y	Y	Y	Y	Y	Y	Y	Y	Y	Y	Y	Y	Y	Y	Y	N	Y	Y	Y
6015-Reanalysis	25		Y	Y	Y	Y	Y	Y	Y	Y	Y	Y	Y	Y	Y	Y	Y	Y	Y	Y	Y	Y	Y	Y	N	Y	Y	Y
6016-Enrollment	18	4	N	Y	Y	Y	Y	N	N	Y	Y	N	Y	Y	Y	N	Y	Y	Y	Y	Y	Y	Y	Y	N	Y	N	N
6016-Reanalysis	22		Y	Y	Y	Y	Y	N	N	Y	Y	Y	Y	Y	Y	Y	Y	Y	Y	Y	Y	Y	Y	Y	N	Y	Y	N
6017-Enrollment	13	9	N	Y	N	N	Y	N	Y	Y	N	Y	Y	Y	N	Y	Y	Y	N	Y	N	Y	Y	N	N	N	N	N
6017-Reanalysis	22		Y	Y	Y	N	Y	N	Y	Y	N	Y	Y	Y	Y	Y	Y	Y	Y	Y	Y	Y	Y	Y	N	Y	Y	Y
6022-Enrollment	17	3	N	Y	N	N	Y	N	Y	Y	Y	Y	Y	N	Y	Y	Y	Y	N	Y	Y	Y	Y	N	N	Y	Y	N
6022-Reanalysis	20		N	Y	Y	Y	Y	N	Y	Y	Y	Y	Y	N	Y	Y	Y	Y	N	Y	Y	Y	Y	Y	N	Y	Y	N
6023-Enrollment	15	4	Y	Y	N	N	Y	N	N	Y	Y	N	N	Y	Y	Y	Y	Y	N	Y	Y	N	Y	Y	N	N	Y	N
6023-Reanalysis	19		Y	Y	N	Y	Y	N	N	Y	Y	Y	Y	Y	Y	Y	Y	Y	N	Y	Y	N	Y	Y	N	Y	Y	N
6025-Enrollment	23	0	Y	Y	Y	Y	Y	N	Y	Y	Y	Y	Y	Y	Y	Y	Y	Y	Y	Y	Y	Y	Y	Y	N	Y	Y	N
6025-Reanalysis	23		Y	Y	Y	Y	Y	N	Y	Y	Y	Y	Y	Y	Y	Y	Y	Y	Y	Y	Y	Y	Y	Y	N	Y	Y	N

6027-Enrollment	21	1	Y	Y	N	Y	Y	N	N	Y	Y	Y	Y	N	Y	Y	Y	Y	Y	Y	Y	Y	Y	Y	N	Y	Y	Y
6027-Reanalysis	22		Y	Y	Y	Y	Y	N	N	Y	Y	Y	Y	N	Y	Y	Y	Y	Y	Y	Y	Y	Y	Y	N	Y	Y	Y
6032-Enrollment	15	7	Y	Y	N	N	Y	N	N	Y	Y	N	Y	N	Y	Y	Y	Y	N	Y	Y	N	Y	N	N	Y	Y	N
6032-Reanalysis	22		Y	Y	Y	Y	Y	N	Y	Y	Y	Y	Y	N	Y	Y	Y	Y	Y	Y	Y	Y	Y	Y	N	Y	Y	N
6033-Enrollment	24	0	Y	Y	Y	Y	Y	N	Y	Y	Y	Y	Y	Y	Y	Y	Y	Y	Y	Y	Y	Y	Y	Y	N	Y	Y	Y
6033-Reanalysis	24		Y	Y	Y	Y	Y	N	Y	Y	Y	Y	Y	Y	Y	Y	Y	Y	Y	Y	Y	Y	Y	Y	N	Y	Y	Y
6034-Enrollment	18	2	Y	Y	N	Y	Y	N	Y	Y	Y	N	Y	N	Y	Y	Y	Y	N	Y	Y	N	Y	Y	N	Y	Y	N
6034-Reanalysis	20		Y	Y	Y	Y	Y	N	Y	Y	Y	N	Y	N	Y	Y	Y	Y	N	Y	Y	Y	Y	Y	N	Y	Y	N
6035-Enrollment	20	2	N	Y	Y	Y	Y	N	Y	Y	N	Y	Y	N	Y	Y	Y	Y	Y	Y	Y	Y	Y	Y	Y	Y	N	N
6035-Reanalysis	22		Y	Y	Y	Y	Y	N	Y	Y	N	Y	Y	N	Y	Y	Y	Y	Y	Y	Y	Y	Y	Y	Y	Y	Y	N
6036-Enrollment	16	1	N	Y	Y	Y	Y	N	N	Y	Y	N	Y	N	Y	Y	Y	Y	N	Y	Y	N	Y	Y	N	N	Y	N
6036-Reanalysis	17		N	Y	Y	Y	Y	N	Y	Y	Y	N	Y	N	Y	Y	Y	Y	N	Y	Y	N	Y	Y	N	N	Y	N
6037-Enrollment	11	5	N	Y	N	N	Y	N	Y	Y	N	N	Y	N	Y	N	Y	Y	N	Y	N	N	Y	Y	N	N	N	N
6037-Reanalysis	16		N	Y	N	N	Y	N	Y	Y	N	Y	Y	N	Y	Y	Y	Y	Y	Y	N	Y	Y	Y	N	Y	N	N
6038-Enrollment	23	0	Y	Y	Y	Y	Y	N	Y	Y	Y	Y	Y	N	Y	Y	Y	Y	Y	Y	Y	Y	Y	Y	N	Y	Y	Y
6038-Reanalysis	23		Y	Y	Y	Y	Y	N	Y	Y	Y	Y	Y	N	Y	Y	Y	Y	Y	Y	Y	Y	Y	Y	N	Y	Y	Y
6040-Enrollment	17	1	Y	Y	Y	N	Y	N	N	Y	N	Y	Y	Y	Y	Y	Y	Y	Y	Y	N	N	Y	Y	N	Y	N	N
6040-Reanalysis	18		Y	Y	Y	Y	Y	N	N	Y	N	Y	Y	Y	Y	Y	Y	Y	Y	Y	N	N	Y	Y	N	Y	N	N
6043-Enrollment	21	1	Y	Y	Y	Y	Y	N	Y	Y	Y	Y	Y	N	Y	Y	Y	Y	Y	Y	Y	Y	Y	Y	N	Y	N	N
6043-Reanalysis	22		Y	Y	Y	Y	Y	N	Y	Y	Y	Y	Y	N	Y	Y	Y	Y	Y	Y	Y	Y	Y	Y	N	Y	Y	N
6046-Enrollment	12	1	N	Y	N	N	Y	N	N	Y	N	N	N	N	Y	Y	Y	Y	N	Y	N	N	Y	Y	N	N	Y	Y
6046-Reanalysis	13		N	Y	N	N	Y	N	N	Y	N	N	Y	N	Y	Y	Y	Y	N	Y	N	N	Y	Y	N	N	Y	Y
6047-Enrollment	18	6	N	Y	Y	Y	Y	N	Y	Y	Y	Y	Y	N	Y	Y	Y	Y	Y	Y	N	N	Y	N	N	N	Y	Y
6047-Reanalysis	24		Y	Y	Y	Y	Y	N	Y	Y	Y	Y	Y	Y	Y	Y	Y	Y	Y	Y	Y	Y	Y	Y	N	Y	Y	Y
6049-Enrollment	25	0	Y	Y	Y	Y	Y	Y	Y	Y	Y	Y	Y	Y	Y	Y	Y	Y	Y	Y	Y	Y	Y	Y	N	Y	Y	Y
6049-Reanalysis	25		Y	Y	Y	Y	Y	Y	Y	Y	Y	Y	Y	Y	Y	Y	Y	Y	Y	Y	Y	Y	Y	Y	N	Y	Y	Y
605N-Enrollment	21	3	Y	Y	Y	Y	Y	Y	N	Y	Y	Y	Y	N	Y	Y	Y	Y	Y	Y	Y	Y	Y	Y	N	Y	N	N
6050-Reanalysis	24		Y	Y	Y	Y	Y	Y	Y	Y	Y	Y	Y	N	Y	Y	Y	Y	Y	Y	Y	Y	Y	Y	N	Y	Y	Y
6055-Enrollment	24	1	Y	Y	Y	Y	Y	N	Y	Y	Y	Y	Y	Y	Y	Y	Y	Y	Y	Y	Y	Y	Y	Y	N	Y	Y	Y
6055-Reanalysis	25		Y	Y	Y	Y	Y	Y	Y	Y	Y	Y	Y	Y	Y	Y	Y	Y	Y	Y	Y	Y	Y	Y	N	Y	Y	Y
6057-Enrollment	20	0	Y	Y	N	N	Y	N	Y	Y	Y	Y	Y	N	Y	Y	Y	Y	Y	Y	Y	Y	Y	Y	N	Y	Y	N
6057-Reanalysis	20		Y	Y	N	N	Y	N	Y	Y	Y	Y	Y	N	Y	Y	Y	Y	Y	Y	Y	Y	Y	Y	N	Y	Y	N
6058-Enrollment	20	0	N	Y	N	Y	Y	N	Y	Y	Y	Y	Y	Y	Y	Y	Y	Y	Y	Y	Y	Y	Y	Y	N	Y	N	N
6058-Reanalysis	20		N	Y	N	Y	Y	N	Y	Y	Y	Y	Y	Y	Y	Y	Y	Y	Y	Y	Y	Y	Y	Y	N	Y	N	N
6060-Enrollment	22	2	Y	Y	Y	N	Y	Y	Y	Y	Y	Y	Y	Y	Y	Y	Y	Y	Y	Y	Y	Y	Y	Y	N	Y	N	N
6060-Reanalysis	24		Y	Y	Y	Y	Y	Y	Y	Y	Y	Y	Y	Y	Y	Y	Y	Y	Y	Y	Y	Y	Y	Y	N	Y	Y	N

6061-Enrollment	19	1	Y	Y	Y	Y	Y	N	Y	Y	N	Y	Y	N	Y	Y	Y	Y	Y	Y	N	Y	Y	Y	N	Y	N	N
6061-Reanalysis	20		Y	Y	Y	Y	Y	N	Y	Y	N	Y	Y	Y	Y	Y	Y	Y	Y	Y	N	Y	Y	Y	N	Y	N	N
6062-Enrollment	23	0	Y	Y	Y	Y	Y	N	Y	Y	Y	Y	Y	Y	Y	Y	Y	Y	Y	Y	Y	Y	Y	Y	N	Y	Y	N
6062-Reanalysis	23		Y	Y	Y	Y	Y	N	Y	Y	Y	Y	Y	Y	Y	Y	Y	Y	Y	Y	Y	Y	Y	Y	N	Y	Y	N
6064-Enrollment	20	2	Y	Y	N	N	Y	N	Y	Y	Y	Y	Y	N	Y	Y	Y	Y	Y	Y	Y	N	Y	Y	N	Y	Y	Y
6064-Reanalysis	22		Y	Y	Y	N	Y	N	Y	Y	Y	Y	Y	N	Y	Y	Y	Y	Y	Y	Y	Y	Y	Y	N	Y	Y	Y
6065-Enrollment	17	0	Y	Y	Y	N	Y	N	Y	Y	Y	N	Y	N	Y	Y	Y	Y	N	Y	Y	N	Y	N	N	N	Y	Y
6065-Reanalysis	17		Y	Y	Y	N	Y	N	Y	Y	Y	N	Y	N	Y	Y	Y	Y	N	Y	Y	N	Y	N	N	N	Y	Y
6066-Enrollment	18	2	Y	Y	Y	Y	Y	N	Y	Y	Y	Y	Y	N	Y	Y	Y	Y	Y	Y	N	N	Y	N	N	Y	N	N
6066-Reanalysis	20		Y	Y	Y	Y	Y	N	Y	Y	Y	Y	Y	N	Y	Y	Y	Y	Y	Y	Y	N	Y	Y	N	Y	N	N
6068-Enrollment	24	0	Y	Y	Y	Y	Y	N	Y	Y	Y	Y	Y	Y	Y	Y	Y	Y	Y	Y	Y	Y	Y	Y	N	Y	Y	Y
6068-Reanalysis	24		Y	Y	Y	Y	Y	N	Y	Y	Y	Y	Y	Y	Y	Y	Y	Y	Y	Y	Y	Y	Y	Y	N	Y	Y	Y
6069-Enrollment	9	10	N	Y	Y	N	N	N	N	N	N	Y	Y	N	N	Y	Y	Y	N	Y	N	N	Y	N	N	N	N	N
6069-Reanalysis	19		Y	Y	Y	N	Y	N	N	Y	N	Y	Y	Y	Y	Y	Y	Y	Y	Y	Y	Y	Y	Y	N	Y	N	N
6070-Enrollment	12	1	N	Y	N	N	Y	N	N	Y	Y	N	Y	N	Y	Y	Y	Y	N	Y	N	N	Y	N	N	Y	N	N
6070-Reanalysis	13		N	Y	N	N	Y	N	N	Y	Y	Y	Y	N	Y	Y	Y	Y	N	Y	N	N	Y	N	N	Y	N	N
6071-Enrollment	19	0	Y	Y	Y	Y	Y	Y	N	Y	N	Y	Y	N	Y	Y	Y	Y	Y	Y	N	Y	Y	Y	N	Y	N	N
6071-Reanalysis	19		Y	Y	Y	Y	Y	Y	N	Y	N	Y	Y	N	Y	Y	Y	Y	Y	Y	N	Y	Y	Y	N	Y	N	N
6072-Enrollment	22	0	Y	Y	Y	Y	Y	Y	Y	Y	Y	N	Y	N	Y	Y	Y	Y	Y	Y	Y	Y	Y	Y	N	Y	Y	N
6072-Reanalysis	22		Y	Y	Y	Y	Y	Y	Y	Y	Y	N	Y	N	Y	Y	Y	Y	Y	Y	Y	Y	Y	Y	N	Y	Y	N
6073-Enrollment	20	2	N	Y	Y	Y	Y	N	Y	Y	Y	Y	Y	N	Y	Y	Y	Y	Y	Y	Y	Y	Y	Y	N	N	Y	N
6073-Reanalysis	22		Y	Y	Y	Y	Y	N	Y	Y	Y	Y	Y	N	Y	Y	Y	Y	Y	Y	Y	Y	Y	Y	N	Y	Y	N
6074-Enrollment	17	5	N	N	N	N	Y	N	N	Y	Y	Y	Y	Y	Y	Y	Y	Y	Y	Y	Y	N	Y	Y	N	Y	Y	N
6074-Reanalysis	22		N	Y	Y	Y	Y	N	Y	Y	Y	Y	Y	Y	Y	Y	Y	Y	Y	Y	Y	Y	Y	Y	N	Y	Y	N
6075-Enrollment	17	0	N	Y	N	N	Y	N	N	Y	Y	Y	Y	N	Y	Y	Y	Y	N	Y	Y	Y	Y	Y	N	N	Y	Y
6075-Reanalysis	17		N	Y	N	N	Y	N	N	Y	Y	Y	Y	N	Y	Y	Y	Y	N	Y	Y	Y	Y	Y	N	N	Y	Y
6076-Enrollment	20	4	N	Y	Y	Y	Y	N	Y	Y	N	Y	Y	N	Y	Y	Y	Y	Y	Y	Y	Y	Y	Y	N	Y	Y	N
6076-Reanalysis	24		Y	Y	Y	Y	Y	Y	Y	Y	Y	Y	Y	Y	Y	Y	Y	Y	Y	Y	Y	Y	Y	Y	N	Y	Y	N
6077-Enrollment	18	1	Y	Y	N	N	Y	Y	Y	Y	Y	Y	Y	Y	Y	N	Y	Y	Y	Y	Y	N	Y	Y	N	N	N	N
6077-Reanalysis	19		Y	Y	N	N	Y	Y	Y	Y	Y	Y	Y	Y	Y	Y	Y	Y	Y	Y	Y	N	Y	Y	N	N	N	N
6080-Enrollment	20	1	Y	Y	N	Y	Y	N	N	Y	N	Y	Y	N	Y	Y	Y	Y	Y	Y	Y	Y	Y	Y	N	Y	Y	Y
6080-Reanalysis	21		Y	Y	N	Y	Y	N	Y	Y	N	Y	Y	N	Y	Y	Y	Y	Y	Y	Y	Y	Y	Y	N	Y	Y	Y
6081-Enrollment	10	10	Y	N	N	N	N	N	N	Y	Y	N	N	N	Y	N	N	Y	N	N	Y	Y	Y	Y	N	Y	N	N
6081-Reanalysis	20		Y	Y	N	Y	Y	N	N	Y	Y	Y	Y	N	Y	Y	Y	Y	Y	Y	Y	Y	Y	Y	N	Y	Y	N

Supplementary Table 4: HPO terms contributing to the diagnostic variant's shortlist ranking in Case 6009. The subset of HPO terms that contributed to Moon's ranking of the diagnostic *IGF2* variant at enrollment and reanalysis are shown.

HPO term	Term Name
Present at initial analysis	
HP:0001629	Ventricular septal defect
HP:0001643	Patent ductus arteriosus
HP:0011682	Perimembranous ventricular septal defect
HP:0001159	Syndactyly
HP:0001508	Failure to thrive
HP:0011470	Nasogastric tube feeding in infancy
HP:0040288	Nasogastric tube feeding
HP:0001999	Abnormal facial shape
HP:0001824	Weight loss
HP:0011968	Feeding difficulties
HP:0000274	Small face
HP:0000347	Micrognathia
HP:0000278	Retrognathia
HP:0000995	Melanocytic nevus
HP:0003764	Nevus
HP:0006101	Finger syndactyly
HP:0100814	Blue nevus
Present only at reanalysis	
HP:0001263	Global developmental delay
HP:0001518	Small for gestational age
HP:0011649	Patent ductus arteriosus after premature birth
HP:0001270	Motor delay
HP:0001290	Generalized hypotonia
HP:0000283	Broad face
HP:0004322	Short stature
HP:0001371	Flexion contracture
HP:0003121	Limb joint contracture
HP:0012785	Flexion contracture of finger
HP:0030044	Flexion contracture of digit
HP:0100360	Contractures of the joints of the upper limbs
HP:0200055	Small hand

Supplementary Table 5: HPO terms contributing to the diagnostic variants' shortlist ranking in Case 6033. The subset of HPO terms that contributed to Moon's ranking of the diagnostic *ERCC6* variants at enrollment and reanalysis are shown.

HPO term	Term Name
Present at initial analysis	
HP:0001508	Failure to thrive
HP:0012444	Brain atrophy
HP:0002090	Pneumonia
HP:0002373	Febrile seizures
HP:0011951	Aspiration pneumonia
HP:0001337	Tremor
HP:0000822	Hypertension
HP:0004322	Short stature
HP:0011952	Acute aspiration pneumonia
HP:0002345	Action tremor
HP:0030186	Kinetic tremor
HP:0001251	Ataxia
HP:0030188	Tremor by anatomical site
HP:0001649	Tachycardia
HP:0200085	Limb tremor
HP:0006532	Recurrent pneumonia
HP:0001288	Gait disturbance
HP:0002141	Gait imbalance
HP:0002066	Gait ataxia
HP:0002317	Unsteady gait
HP:0030187	Titubation
HP:0001371	Flexion contracture
HP:0002078	Truncal ataxia
HP:0002283	Global brain atrophy
HP:0000958	Dry skin
HP:0001250	Seizures
HP:0001919	Acute kidney injury
HP:0007369	Atrophy/Degeneration affecting the cerebrum
HP:0011675	Arrhythmia
HP:0000639	Nystagmus
HP:0010783	Erythema
HP:0000486	Strabismus
HP:0000540	Hypermetropia

HP:0002346	Head tremor
HP:0010877	Monocular strabismus
HP:0000093	Proteinuria
HP:0000490	Deeply set eye
HP:0001525	Severe failure to thrive
HP:0002355	Difficulty walking
HP:0011703	Sinus tachycardia
HP:0000252	Microcephaly
HP:0002070	Limb ataxia
HP:0002378	Hand tremor
HP:0002527	Falls
HP:0002540	Inability to walk
HP:0009900	Unilateral deafness
HP:0001041	Facial erythema
HP:0001510	Growth delay
HP:0001662	Bradycardia
HP:0001962	Palpitations
HP:0002135	Basal ganglia calcification
HP:0002322	Resting tremor
HP:0002415	Leukodystrophy
HP:0003121	Limb joint contracture
HP:0007371	Corpus callosum atrophy
HP:0040323	Erythema of the eyelids
Present only at reanalysis	
HP:0002240	Hepatomegaly
HP:0010542	Vestibular nystagmus
HP:0012593	Nephrotic range proteinuria
HP:0012622	Chronic kidney disease
HP:0001744	Splenomegaly
HP:0012594	Microalbuminuria
HP:0000670	Carious teeth
HP:0002059	Cerebral atrophy
HP:0012624	Stage 2 chronic kidney disease
HP:0000238	Hydrocephalus
HP:0000488	Retinopathy
HP:0002123	Generalized myoclonic seizures
HP:0100963	Hyperesthesia
HP:0000083	Renal insufficiency
HP:0000875	Episodic hypertension
HP:0003510	Severe short stature
HP:0012592	Albuminuria
HP:0012595	Mild proteinuria
HP:0012596	Moderate proteinuria
