# Supplementary material for: Partially automated whole-genome sequencing reanalysis of previously undiagnosed pediatric patients can efficiently yield new diagnoses
Source: NPJ Genom Med. 2020 Aug 11;5:33. doi: 10.1038/s41525-020-00140-1 (PMC7419288; doi:10.1038/s41525-020-00140-1)
Supplement: Supplementary file 2 — Reporting Summary [file 41525_2020_140_MOESM2_ESM.pdf]

nature research

Corresponding author(s): Kiely N. James

Last updated by author(s): May 28, 2020

Reporting Summary

Nature Research wishes to improve the reproducibility of the work that we publish. This form provides structure for consistency and transparency in reporting. For further information on Nature Research policies, see our Editorial Policies and the Editorial Policy Checklist.

Statistics

For all statistical analyses, confirm that the following items are present in the figure legend, table legend, main text, or Methods section.

n/a	Confirmed	
☐	☒	The exact sample size (*n*) for each experimental group/condition, given as a discrete number and unit of measurement
☒	☐	A statement on whether measurements were taken from distinct samples or whether the same sample was measured repeatedly
☐	☒	The statistical test(s) used AND whether they are one- or two-sided<br>*Only common tests should be described solely by name; describe more complex techniques in the Methods section.*
☒	☐	A description of all covariates tested
☒	☐	A description of any assumptions or corrections, such as tests of normality and adjustment for multiple comparisons
☐	☒	A full description of the statistical parameters including central tendency (e.g. means) or other basic estimates (e.g. regression coefficient) AND variation (e.g. standard deviation) or associated estimates of uncertainty (e.g. confidence intervals)
☐	☒	For null hypothesis testing, the test statistic (e.g. *F*, *t*, *r*) with confidence intervals, effect sizes, degrees of freedom and *P* value noted<br>*Give P values as exact values whenever suitable.*
☒	☐	For Bayesian analysis, information on the choice of priors and Markov chain Monte Carlo settings
☒	☐	For hierarchical and complex designs, identification of the appropriate level for tests and full reporting of outcomes
☐	☒	Estimates of effect sizes (e.g. Cohen's *d*, Pearson's *r*), indicating how they were calculated

Our web collection on statistics for biologists contains articles on many of the points above.

Software and code

Policy information about availability of computer code

Data collection	This information was collated from several subsections within the Materials and Methods section.<br><br>Alignment and nucleotide variant calling was performed using the DRAGEN hardware and software platform (version 2.1.5). VCF files from DRAGEN were annotated and analyzed in Opal Clinical versions 4.20-4.28. Human Phenotype Ontology (HPO) terms were re-extracted from patient records at the time of reanalysis, using CLiX ENRICH (Clinithink, Alpharetta, GA) as previously described. . Study participant VCF files, together with HPO term lists from CLiX ENRICH, were uploaded to Moon (Diploid, Leuven, Belgium) (Version details: ClinVar: 2018-04-29; dbNSFP: 3.5; dbSNP: 150; dbscSNV: 1.1; Apollo: 2018-05-03; Ensembl: 37; gnomAD: 2.0.1; HPO: 2017-10-05; Moon: 2.0.3) for automated, phenotype-driven variant analysis, as previously described12. For comparisons of Moon variant shortlists with HPO terms drawn from EHRs at enrollment or reanalysis, a newer version of Moon (Version details: ClinVar: 2019-03-11; dbNSFP: 3.5; dbSNP: 150; dbscSNV: 1.1; Apollo: 2019-03-18; Ensembl: 37; gnomAD: 2.0.1; HPO: 2019-02-12; KB: 2019-04-03; DGV: 2016-03-01; dbVar: 2018-06-24; Mitomap: 2019-01-14; Mitimpact: 2.9.1; Mastermind: 2018-11-26; Moon: 3.0.3) was used. Copy number analysis was performed as previously described17. Briefly, the read pair based tool, Manta, was used to detect smaller CNVs while the coverage based caller, CNVnator, was used to detect larger CNVs.
Data analysis	Nonparametric Spearman correlations, Wilcoxon signed rank tests and corresponding two-tailed p-values were calculated using Prism (version 6.0h, GraphPad, La Jolla, CA). The 95% confidence interval (CI) for the proportion of new diagnoses made upon reanalysis was calculated using the binomial exact (Clopper-Pearson) method

For manuscripts utilizing custom algorithms or software that are central to the research but not yet described in published literature, software must be made available to editors and reviewers. We strongly encourage code deposition in a community repository (e.g. GitHub). See the Nature Research guidelines for submitting code & software for further information.

Data

Policy information about availability of data

All manuscripts must include a data availability statement. This statement should provide the following information, where applicable:

- Accession codes, unique identifiers, or web links for publicly available datasets
- A list of figures that have associated raw data
- A description of any restrictions on data availability

The variant list data that were used in reanalysis are available as Supplementary Data. The sequencing data that the variant lists are drawn from are available from the corresponding author upon reasonable request and completion of a data use agreement, subject to the limitations of the informed consent documents for each subject.

Field-specific reporting

Please select the one below that is the best fit for your research. If you are not sure, read the appropriate sections before making your selection.

☒ Life sciences ☐ Behavioural & social sciences ☐ Ecological, evolutionary & environmental sciences

For a reference copy of the document with all sections, see nature.com/documents/nr-reporting-summary-flat.pdf

Life sciences study design

All studies must disclose on these points even when the disclosure is negative.

Sample size	A sample of 48 negative cases was selected. This was the number that were available for which at least 16 months had passed since analysis.
Data exclusions	There were no data exclusions in this study.
Replication	There was no replication in this study.
Randomization	There was no randomization in this study.
Blinding	There was no blinding in this study.

Reporting for specific materials, systems and methods

We require information from authors about some types of materials, experimental systems and methods used in many studies. Here, indicate whether each material, system or method listed is relevant to your study. If you are not sure if a list item applies to your research, read the appropriate section before selecting a response.

Materials & experimental systems

n/a	Involved in the study	
☒	☐	Antibodies
☒	☐	Eukaryotic cell lines
☒	☐	Palaeontology and archaeology
☒	☐	Animals and other organisms
☐	☒	Human research participants
☒	☐	Clinical data
☒	☐	Dual use research of concern

Methods

n/a	Involved in the study	
☒	☐	ChIP-seq
☒	☐	Flow cytometry
☒	☐	MRI-based neuroimaging

Human research participants

Policy information about studies involving human research participants

Population characteristics

See Table 1:
Age at enrollment
Median: 5months; Range 0.1-238 months
< 1 month 10 (21%)
< 6 months 27 (56%)
Sex
Female 23 (48%)
Male 25 (52%)
Race and Ethnicity
Hispanic/Latino 24 (50%)

	Caucasian 14 (29%)<br>Asian/Pacific Islander 3 (6%)<br>African/African American 2 (4%)<br>Other/Unknown 5 (10%)
Recruitment	Inpatients at RCHSD without etiologic diagnoses, in whom a genetic disorder was possible, were nominated for diagnostic, rapid WGS by diverse clinicians from July 26 2016–April 3 2017.
Ethics oversight	Institutional review board (IRB) at the University of California, San Diego

Note that full information on the approval of the study protocol must also be provided in the manuscript.
